# APOBEC3C‐mediated NF‐κB activation enhances clear cell renal cell carcinoma progression

**DOI:** 10.1002/1878-0261.13721

**Published:** 2024-08-26

**Authors:** Nora Hase, Danny Misiak, Helge Taubert, Stefan Hüttelmaier, Michael Gekle, Marcel Köhn

**Affiliations:** ^1^ Junior Group ‘Non‐Coding RNAs and RBPs in Human Diseases’, Medical Faculty Martin Luther University Halle/Wittenberg Germany; ^2^ Section for Molecular Cell Biology, Institute of Molecular Medicine Martin Luther University Halle/Wittenberg Germany; ^3^ Department of Urology and Pediatric Urology University Hospital Erlangen, Friedrich Alexander University Erlangen/Nürnberg Germany; ^4^ Julius‐Bernstein‐Institute of Physiology Martin Luther University Halle/Wittenberg Germany

**Keywords:** APOBEC3C, ccRCC, NF‐κB, RBP

## Abstract

Renowned as the predominant form of kidney cancer, clear cell renal cell carcinoma (ccRCC) exhibits susceptibility to immunotherapies due to its specific expression profile as well as notable immune cell infiltration. Despite this, effectively treating metastatic ccRCC remains a significant challenge, necessitating a more profound comprehension of the underlying molecular mechanisms governing its progression. Here, we unveil that the enhanced expression of the RNA‐binding protein DNA dC → dU‐editing enzyme APOBEC‐3C (APOBEC3C; also known as A3C) in ccRCC tissue and ccRCC‐derived cell lines serves as a catalyst for tumor growth by amplifying nuclear factor‐kappa B (NF‐κB) activity. By employing RNA‐sequencing and cell‐based assays in ccRCC‐derived cell lines, we determined that A3C is a stress‐responsive factor and crucial for cell survival. Furthermore, we identified that A3C binds and potentially stabilizes messenger RNAs (mRNAs) encoding positive regulators of the NF‐κB pathway. Upon A3C depletion, essential subunits of the NF‐κB family are abnormally restrained in the cytoplasm, leading to deregulation of NF‐κB target genes. Our study illuminates the pivotal role of A3C in promoting ccRCC tumor development, positioning it as a prospective target for future therapeutic strategies.

## Graphical abstract

The authors discovered that expression of the RNA‐binding protein APOBEC3C (A3C) promotes clear cell renal cell carcinoma (ccRCC) growth by amplifying nuclear factor‐kappa B (NF‐κB) activity. A3C‐bound mRNAs of NF‐κB regulators are stabilized, which promotes NF‐κB‐subunit shuttling and transcriptional activity. This study highlights A3C's role in enhancing ccRCC progression, positioning it as a target for therapeutic strategies.

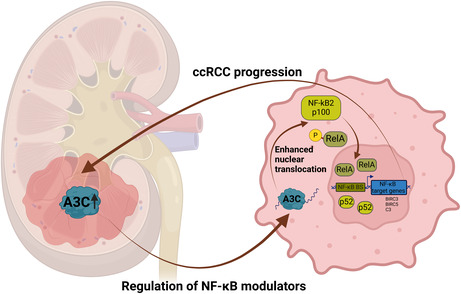

**Abbreviations**
A3C: APOBEC3C, apolipoprotein B mRNA editing enzyme catalytic subunit 3C
ccRCC: clear cell renal cell carcinoma
chrRCC: chromophobe renal cell carcinoma
CRISPR: clustered regularly interspaced short palindromic repeats
DEG: differentially expressed genes
FC: fold change
GSEA: gene set enrichment analysis
HA: high attachment
HD: high density
HIF: hypoxia‐inducible factor
IκB: inhibitory kappa B
KIRC: kidney renal clear cell carcinoma
KO: knockout
LA: low attachment
LD: low density
NF‐κB: nuclear factor‐kappa B
NMD: nonsense‐mediated mRNA decay
NT: normal tissue
papRCC: papillary renal cell carcinoma
RCC: renal cell carcinoma
Rec: recovery
RIP: RNA (co)‐immunoprecipitation
RNA‐seq: RNA sequencing
RPKM: reads per kilobase of transcript per million mapped reads
RT‐qPCR: real time quantitative PCR
TCGA: the cancer genome atlas
UTR: untranslated region
WB: western blot

## 1 Introduction

Renal cell carcinoma (RCC) is among the 10 most common cancers worldwide [1] and affects nearly 300 000 individuals every year [2]. RCC comprises several different cancer subtypes that derive from renal tubular epithelial cells [3]. The clear cell renal cell carcinoma (ccRCC) is the most common subtype, accounting for approximately 75% of all kidney cancers [4]. Other subtypes include the papillary renal cell carcinoma (papRCC) and the chromophobe renal cell carcinoma (chrRCC), which account for 15–20% [5] and ~ 5% [6] of all RCCs, respectively. In total, approximately 50% [7] of ccRCCs are characterized by inactivation of the tumor suppressor gene *VHL* either by local genetic or epigenetic changes or the loss of chromosome 3p [8, 9, 10]. Consequently, targets of the pVHL containing E3 ubiquitin ligase complex are no longer efficiently degraded, for example, hypoxia‐inducible factors (HIFs) [11]. The accumulation of HIF transcription factors results in tumor progression by activating gene expression involved in cancer development such as angiogenesis, migration, and invasion [12, 13]. As previously shown, the loss of *VHL* can activate nuclear factor‐κB (NF‐κB) in RCC cells [14, 15] and increased NF‐κB activity is associated with cancer progression in RCC patients, highlighting that the NF‐κB signaling pathway is a key regulator of immune responses and inflammation [16, 17, 18, 19]. In addition, it is well established that chronic inflammation is a hallmark of cancer in general [20, 21, 22]. Considering that RCC is a tumor type that is strongly influenced by immunomodulatory and inflammation processes, it was previously shown that targeting the NF‐κB pathway blocks RCC tumor growth *in vivo* [23, 24, 25, 26, 27, 28].

The NF‐κB family of transcription factors encompasses the subunits NF‐κB1 (p50/p105), NF‐κB2 (p52/p100), RelA (p65), RelB and c‐Rel [29]. These individual proteins can form several combinations of homo‐ or heterodimers to form the active NF‐κB transcription unit [30]. Activation of the subunits occurs by a complex regulatory network and involves the degradation of inhibitory proteins upon phosphorylation [31, 32]. The phosphorylation cascade is stimulated by diverse factors including viral pathogens, cytokines, or growth factors [33, 34]. Subsequently, NF‐κB complexes are translocated to the nucleus, bind to genomic κB‐sites and induce synthesis of various proliferative, anti‐apoptotic and angiogenic genes as well as genes involved in metastasis [35].

While transcriptional networks have been well investigated in ccRCC, the role of post‐transcriptional regulation, especially by RNA‐binding proteins (RBPs), has barely been addressed. RBPs are considered one of the major factors influencing post‐transcriptional gene expression [36]. However, only a few studies specify the role of dysregulated RBPs in ccRCC [37, 38, 39, 40]. As an example of the RBP family, members of the APOBEC3 (apolipoprotein B mRNA editing enzyme catalytic polypeptide‐like 3) protein family have been reported to be upregulated in ccRCC [41, 42, 43]. APOBEC3 (A3) proteins belong to the group of cytidine deaminases that can edit DNA and RNA to potentially induce mutations [44, 45]. In total, there are seven members of the A3 family in humans (A3A, A3B, A3C, A3D, A3F, A3G, and A3H [46]). The main function of A3 enzymes proposed so far is the inhibition of retroviral replication, a major part of the innate immune response [47, 48, 49]. However, deregulation of A3 proteins is considered to influence tumorigenesis by introducing somatic mutations in oncogenes or tumor suppressor genes due to their deaminase activity [50]. Especially A3B has been identified as the main source of genomic C‐to‐T mutations upon upregulation in many cancers, for example, breast cancer [51, 52]. In contrast to A3B, which is almost exclusively located in the nucleus, A3C is localized in the cytoplasm [53, 54, 55, 56], suggesting a molecular function of A3C beyond inducing DNA mutations. In this study, we provide new insight into the molecular role of A3C in ccRCC, assessing the associated signaling pathways and potential drug based inhibition in the future.

## 2 Materials and methods

### 2.1 Cell culture

Human renal cell carcinoma cells (786‐O [RRID:CVCL_1051], 769‐P [RRID:CVCL_1050], A‐704 [RRID:CVCL_1065], ACHN [RRID:CVCL_1067]) and immortalized normal kidney cells (HEK293 [RRID:CVCL_0045] and RPTEC/TERT1 [RRID:CVCL_K278]) were obtained from the American Type Culture Collection (ATCC, Manassas, VA, USA). Cells were regularly checked for mycoplasma contamination by PCR. 786‐O and 769‐P cells were maintained in RPMI 1640 medium (Gibco, Thermo Fisher Scientific, Waltham, MA, USA) supplemented with 10% FBS (Cat #P303031; Pan Biotech, Aidenbach, Germany). A‐704, ACHN, and HEK293 cells were maintained in high‐glucose (4.5 g·L^−1^) DMEM (Gibco, Thermo Fisher Scientific) supplemented with GlutaMAX supplement (Gibco, Thermo Fisher Scientific) and 10% FBS. RPTEC/TERT1 cells were cultured in DMEM supplemented with hTERT Immortalized RPTEC Growth Kit (ATCC). All cells were incubated in 5% CO_2_ at 37 °C in a humidified atmosphere. All cell lines have been tested for their identity within the past 3 years by using the Eurofins Genomics CLA service.

### 2.2 Cell transfection (DNA or siRNA) and stable cell clones

One day prior to transfection, 1.5 × 10^5^ cells for 786‐O and 2 × 10^5^ cells for 769‐P were seeded in a 6‐well plate. For siRNA‐mediated gene silencing, cells were transfected with 50 pmol of the indicated siRNA pool (Table S2) using Lipofectamine RNAiMAX (Thermo Fisher Scientific) according to manufacturer's protocol. Plasmid transfection was performed with 2 and 4 μg plasmid DNA (Table S2), respectively, using Lipofectamine 3000 (Thermo Fisher Scientific) or DharmaFECT kb DNA transfection reagent (Horizon Discovery, Cambridge, UK) according to the instructions of the manufacturers. One day after transfection, the medium was changed or cells were treated for further experiments.

For CRISPR/Cas9‐mediated genomic deletion in the *A3C* locus, 786‐O cells were co‐transfected with two CRISPR sgRNA‐encoding plasmids (pSG‐sgRNA3‐A3C#1‐RFP and pSG‐sgRNA3‐A3C#3‐RFP) and a Cas9 nuclease‐encoding plasmid (pCDNA3.1‐Cas9‐GFP; Table S2) as described above. Transfection efficiency was determined by fluorescence microscopy. Twenty‐four hours post transfection, cell populations were diluted until single cell level in a 24‐well plate and cultured to verify A3C KO by western blotting and sanger sequencing. To generate a stable A3C recovery clone, 786‐O A3C KO cells were transfected with pEGFP‐(C2)‐A3C (Table S2), sorted for GFP‐positive cells using the BD FACS Melody, selected with 500 μg·mL^−1^ geneticin 1 day after sorting and cultured under constant geneticin selection pressure (50 μg·mL^−1^). For stable A3C knockdown clones, 786‐O and 769‐P cells were transfected with pLVX‐shRNA3‐shA3C#3 as described above. For selection, transfected cells were treated with 4 μg·mL^−1^ puromycin 1 day after transfection.

### 2.3 Luciferase reporter assay

Stable cell clones were transfected with pmirGLO‐Promo+Stopp*HindIII‐NF‐κB (Table S2), which comprises five NF‐κB binding elements in the promoter region of the Firefly luciferase gene. Renilla luciferase on the same plasmid served as the normalization control. The activities of Firefly and Renilla luciferases were determined 48 h post transfection by DualGLO (Promega, Madison, WI, USA) according to the manufacturer's protocol.

### 2.4 RNA isolation and quantitative RT‐PCR

Total RNA was extracted using Trizol (Thermo Fisher Scientific) either from cell pellets or primary tissue that was mechanically disrupted in Trizol prior to RNA isolation. Equal amounts (2 μg) of total RNA served as a template for cDNA synthesis by random priming using M‐MLV reverse transcription system (Promega). Quantitative PCR was performed based on SYBRgreen I technology using the ORA qPCR Green ROX L Mix (HighQu, Kraichtal, Germany) on a LightCycler 480 II 384 format system (Roche, Basel, Switzerland). Relative changes of RNA abundance were determined by the ΔΔ*C*_t_ method using ACTB and EEF2 for normalization. For primers, see Table S2.

### 2.5 RNA co‐immunoprecipitation

For RNA co‐immunoprecipitations (RIP), 786‐O A3C Rec cell populations (5 × 10^6^ per condition) were lysed using RIP buffer (10 mm Hepes‐KOH [pH 7.2], 300 mm KCl, 5 mm MgCl_2_, 0.5% NP‐40). The supernatants were incubated with 5 μg anti‐GFP (Roche) or anti‐FLAG (Merck, Darmstadt, Germany) and pre‐washed magnetic Protein G Dynabeads (Life Technologies, Waltham, MA, USA) for 60 min at room temperature on a spinning‐wheel. After three washing steps with RIP buffer, protein–RNA complexes were eluted by RIP buffer supplemented with 1% SDS and incubated at 65 °C for 10 min. Protein enrichment was analyzed by western blotting. Co‐purified RNAs were extracted using TRIZOL and analyzed by RT‐qPCR and NGS.

### 2.6 RNA‐sequencing

For total RNA‐sequencing (RNA‐seq) 300 ng RNA of primary tissue samples (*n* (NT) = 8; *n* (ccRCC) = 8; *n* (papRCC T1) = 3; *n* (papRCC T2) = 3; *n* (chrRCC) = 3), of stable 786‐O CRISPR/Cas9‐mediated A3C KO and Rec clones (A3C Rec) and of 786‐O A3C Rec RIP‐samples were used (*n* = 3 in all cell and RIP samples). Library preparation and sequencing were performed by Novogene (Hong Kong) on an Illumina HiSeq platform. First, low‐quality read ends as well as remaining parts of sequencing adapters were clipped using cutadapt (v1.4; 2.8). Subsequently, reads were aligned to the human genome (UCSC GRCh38/hg38) using hisat2 v2.1.0 [57]. featurecounts (v1.5.3; 2.0) [58] was used for summarizing gene‐mapped reads. Ensembl (GRCh38.89; GRCh38.102) [59] was used for annotations. Differential gene expression (DE) was determined by the R package edger (v3.28.0; 3.34) [60] using TMM (trimmed mean of M values) normalization on raw count data. RNA expression values were obtained as FPKM (fragments per kilobase million mapped reads) values.

### 2.7 Public data, gene set enrichment analyses, and Kaplan–Meier analyses

TCGA‐KIRC/KIRP/KICH‐RNA‐seq data of the kidney cancer cohort were obtained from the GDC portal (https://portal.gdc.cancer.gov/). Gene set enrichment analyses (GSEA) were performed on pre‐ranked lists using the gsea‐software (v3.0) [61] selecting Hallmark gene sets from MSigDB (v6.1) [62]. All protein‐coding genes were ranked according to the fold changes in 786‐O C to A3C KO and A3C KO to A3C Rec determined by RNA‐seq. A permutation number of 1000 was applied, and classical enrichment statistics were elected. For survival analyses, overall survival data of RCC patients was obtained from TCGA data sets for ccRCC (KIRC), papRCC (KIRP), and chrRCC (KICH) patients with median group cutoff. For overall survival based on the expression status of A3C, the graphpad prism software (v9.0, Boston, MA, USA) was used to calculate Kaplan–Meier plot and Hazard ratio (HR) by using the log‐rank (Mantel–Cox) test. For survival analyses based on the expression status of other indicated genes, Kaplan–Meier plots and HR were determined using gepia 2 (http://gepia2.cancer‐pku.cn/#survival).

### 2.8 Protein extraction and western blotting

For total protein extraction, cell pellets or pestled primary tissue samples (Comprehensive Cancer Center tissue biobank of the University Hospital Erlangen) were lysed in total lysis buffer (50 mm Tris–HCl pH 7.4, 50 mm NaCl, 1% SDS, 1 mm MgCl_2_, Turbo nuclease [Jena Bioscience, Jena, Germany, 250 U·μL^−1^]). For phosphorylation analyses, protease/phosphatase inhibitor (Cell Signaling, Danvers, MA, USA) was added to the lysis buffer. The protein concentration of each sample was determined using a colorimetric assay (Bio‐Rad, Hercules, CA, USA) according to manufacturer's protocol. Equal amounts of total protein (40 μg) were separated by NuPAGE Bis‐Tris (4–12%) gels (Thermo Fisher Scientific) and transferred to a nitrocellulose membrane (Amersham, GE Healthcare, Chicago, IL, USA) using the Mini Gel Tank Blotting system (Thermo Fisher Scientific). The membranes were blocked for 1 h at room temperature in phosphate‐buffered saline solution (PBS; 140 mm NaCl, 2.6 mm KCl, 1.5 mm KH_2_PO_4_ and 8 mm Na_2_HPO_4_) containing 5% BSA. Membranes were incubated with primary antibody at 4 °C overnight, washed with PBS supplemented with 0.1% Tween‐20, incubated with secondary antibody at room temperature for 1 h and analyzed using the Odyssey infrared scanner (LI‐COR, Lincoln, NE, USA). Primary and secondary antibodies are summarized in Table S2.

### 2.9 Subcellular fractionation

For fractionation, 1.5 × 10^6^ cells per condition were harvested in fractionation buffer (10 mm Hepes‐KOH [pH 7.2], 150 mm KCl, 5 mm MgCl_2_) supplemented with 130 μg·mL^−1^ digitonin (Sigma‐Aldrich, St. Louis, MO, USA) for 786‐O and 200 μg·mL^−1^ for 769‐P. Subcellular fractions were separated by centrifugation at 1000 ***g*** for 2 min. Supernatant containing the cytoplasmic fraction was transferred to a new tube. The remaining pellet was washed with fractionation buffer supplemented with digitonin and centrifuged at 1000 ***g*** for 2 min. Pellets containing the nuclear fraction were lysed in total lysis buffer (50 mm Tris–HCl pH 7.4, 50 mm NaCl, 1% SDS, 1 mm MgCl_2_, Turbo nuclease [Jena Bioscience, 250 U·μL^−1^]). Further analyses were performed by western blotting as described above. Subcellular fractionation was confirmed by detecting EEF2 in the cytoplasmic and PTB in the nuclear fraction.

### 2.10 Cell viability assays (anoikis resistance and 3D spheroid cell cultures)

For anoikis resistance, 1 × 10^3^ cells were seeded either into flat bottom ultra‐low‐attachment 96‐well plates (Cat #3474; Corning, Corning, NY, USA) or into high‐attachment 96‐well plates (Cat #Z707902; TPP, Trasadingen, Switzerland) in growth medium either supplemented with 1% FBS or 10% FBS. When indicated, siRNA‐mediated knockdown was transfected 24 h prior to seeding; otherwise, stabile cell lines were used. Cells were cultured for 5 days. Cell viability was measured using CellTiter‐Glo (Promega) according to manufacturer's protocol. For 3D spheroid growth, 1 × 10^3^ cells were seeded in 100 μL growth medium into round bottom ultra‐low‐attachment 96‐well plates (Cat #7007; Corning), centrifuged for 3 min at 300 ***g*** and cultured overnight to induce spheroid formation. 3D growth was monitored for 5 days using an Incucyte S3 (Sartorius, Göttingen, Germany) device. Images were analyzed by spheroid segmentation with the incucyte software (Sartorius). Images of the first time point served as normalization control.

### 2.11 Patient tissue samples

The RCC cohort is composed of 38 patient tissue samples (Table S2). The normal tissue samples originated from tumor adjacent tissue in five cases from ccRCC patients, in one case from a collecting duct carcinoma patient, in one case from a chromophobe renal cell carcinoma patient and in one case from an oncocytoma patient. The snap‐frozen tissue samples were obtained from the Comprehensive Cancer Center tissue biobank of the University Hospital Erlangen, Germany. The tumor histology was reviewed by experienced uropathologists, as previously described in Ref. [63]. All patients gave written informed consent. The study was based on the approval of the Ethics Commissions of the University Hospital Erlangen (No. 4607). The samples were collected between 2008 and 2014.

### 2.12 Mouse xenograft studies and animal handling

Immunodeficient athymic nude mice (FOXN1^nu/nu^) were obtained from Charles River (Wilmington, MA, US). For subcutaneous xenograft assays 6 × 10^5^ 786‐O C, A3C KO and A3C Rec cells were harvested in media supplemented with 50% (v/v) matrigel (Sigma‐Aldrich) and injected into both flanks of 6‐week old female mice (16 mice in total). Subcutaneous tumor growth was measured in two diameters with calibers to allow calculation of tumor volume. The volume was calculated using the formula *V* = π/6 × (*l* + *w* + *h*). Mice were sacrificed after 8 weeks, as C and A3C Rec derived tumors reached termination criteria.

Mice were kept according to §11/1/1 TSchG (German animal protection law). The keeping was in alignment with the recommendations of the Federation of Laboratory Animal Science Associations (FELASA). Access to animals was electronically restricted. All necessary hygiene measures were taken into consideration. The animals were kept in individually ventilated cages (IVC) with constant monitoring of climate parameters. Feeding and water were supplied *ad libitum*. Enrichment for animals was provided. The experiments were conducted under the animal license 203.m‐42502‐2‐1625 MLU.

### 2.13 Statistical analyses

All experiments were performed in at least three independent, biological replicates unless otherwise stated. Statistical analyses were performed using graphpad prism software (v9.0). Statistical significance was tested by unpaired, two‐tailed Student's *t* test on equally distributed data or by one‐way ANOVA with Tukey's multiple‐comparison test and is indicated in the diagrams as followed: *P < 0.05; **P < 0.01; ***P < 0.001; ****P < 0.0001. Western blot quantifications are shown as mean ± SD. The box and whiskers plots depict the 5–95 percentile. Bar plots depict mean ± SEM and all outliers are shown.

### 2.14 Study approval

All procedures concerning the patient tissue samples were performed in accordance with the ethical standards established in the 1964 Declaration of Helsinki and its later amendments. All patients gave informed consent. The study was based on the approval of the Ethics Commissions of the University Hospital Erlangen, Germany (No. 4607). All animals were handled in accordance with European Directive (2010/63/EU) and local guidelines of the Martin Luther University Halle/Wittenberg. An independent and certified ethic officer from the Martin Luther University Halle/Wittenberg directly approved procedures.

## 3 Results

### 3.1 A3C is upregulated in ccRCC and correlates with poor survival in RCC

**Fig. 1:**
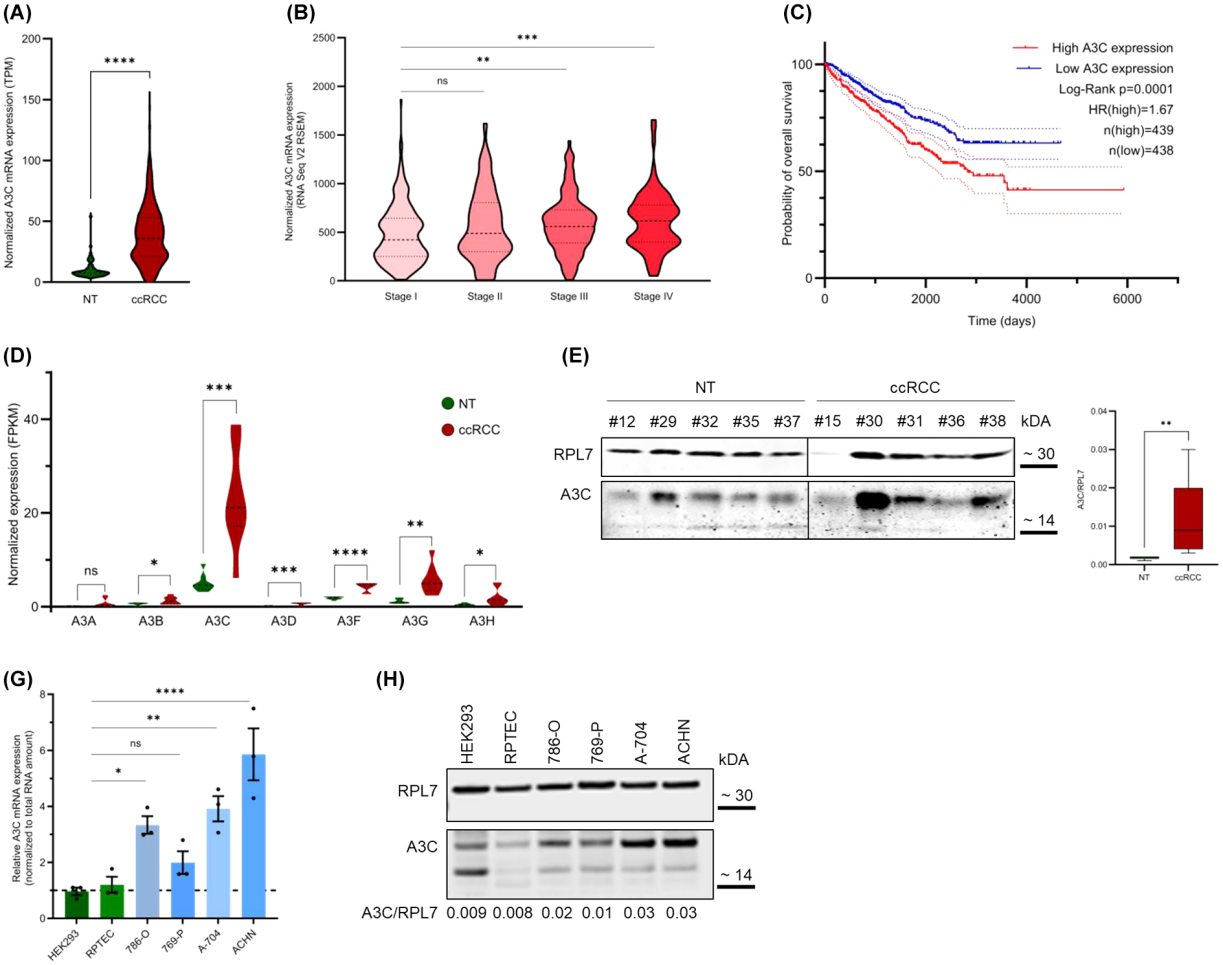
Clinical association of A3C in clear cell renal cell carcinoma (ccRCC). (A) The expression of A3C in ccRCC samples compared to normal tissue (NT) was analyzed. Data were obtained from TCGA (*n* (NT) = 72; *n* (ccRCC) = 507). (B) The relative A3C expression in stage I (*n* = 268), stage II (*n* = 57), stage III (*n* = 123), and stage IV (*n* = 83) ccRCC patients (TCGA data) is depicted as violin plots. (C) Overall survival of 877 RCC patients based on different A3C expression status was investigated by Kaplan–Meier analysis. Data was obtained from TCGA. (D, E) Validation of A3C expression in a separate, small ccRCC cohort. (D) Relative mRNA expression of APOBEC3 family members in ccRCC (*n* = 8) and corresponding NT (*n* = 8) is shown. (E) A3C protein levels in ccRCC primary tissues (*n* = 5 for NT and ccRCC samples) were determined by western blot (WB). The right panel shows quantification of the A3C levels normalized to ribosomal protein L7 (RPL7). The whiskers represent the minimal and maximal values. (F, G) A3C expression was quantified in RCC cell lines (786‐O, 769‐P, A‐704, and ACHN) compared to normal kidney cell lines (HEK293 and RPTEC/TER1) at mRNA (F) and protein (G) level. *P < 0.05; **P < 0.01; ***P < 0.001; ****P < 0.0001 by unpaired, two‐tailed Student's *t* test (A, B, D), two‐tailed Mann–Whitney test (E) or one‐way ANOVA with Tukey's multiple‐comparison test (F). The Kaplan–Meier curve was calculated using the log‐rank (Mantel–Cox) test (C). Data is representative of three independent experiments (mean ± SEM in F).

Initial investigation of a kidney renal clear cell carcinoma (KIRC cohort, TCGA) data set revealed a substantial upregulation of A3C expression (*P* < 0.0001; Fig. 1A). Further in‐depth analyses of A3C expression indicated pronounced abundance of A3C in advanced disease stages, TNM‐3 (*P* = 0.0014) and TNM‐4 (*P* = 0.0004), suggesting elevated expression during disease progression (Fig. 1B). To evaluate the putative prognostic value of A3C expression, we used the overall survival data of patients from the TCGA. We divided the samples into two equal groups based on A3C levels. Patients with higher A3C levels showed poorer overall survival (*P* = 0.0001) and a higher probability of succumbing to the disease (HR = 1.67; Fig. 1C). These results indicate that A3C exhibits prognostic value in ccRCC.

To confirm high A3C expression levels in ccRCC samples, we analyzed a separate, smaller RCC cohort. Our initial analysis of the transcriptomic profiles of ccRCC and normal tissue (NT) samples revealed that all A3 family members, except for A3A, showed significantly increased expression in ccRCC compared to NT (Fig. 1D and Fig. S1A). However, among the A3 family members, A3C mRNA displayed the highest upregulation, confirming the results obtained from the TCGA cohort. Next, we verified also at the protein level a significant increase of A3C in ccRCC tumor samples (Fig. 1E). We further investigated A3C expression levels in smaller cohorts of other RCC subtypes (Fig. S1B) to evaluate if clinical significance of A3C is restricted to the ccRCC subtype. The papillary subtype, either type 1 or 2, revealed upregulated A3C levels similar to ccRCC, whereas the chromophobe subtype of RCC did not show increased A3C abundance.

In order to investigate the function of A3C in more detail, we employed RCC model cell systems. These cell lines were derived from the clear cell subtype of RCC (786‐O, 769‐P, and A‐704), the papillary subtype (ACHN) [64] or generated by immortalization of non‐transformed kidney cells (HEK293, RPTEC/TERT1). All these cell models expressed A3C but showed markedly distinct abundance of A3C protein and mRNA (Fig. 1F,G). A3C expression was substantially enhanced (~ 3‐fold) in tumor cells derived from clear cells as well as papillary subtypes of renal cancer in comparison to transformed cells and 769‐P, supporting findings in primary diseases. In sum, these findings indicate that A3C is upregulated in renal cancer and associated with diseases progression and adverse disease outcome.

### 3.2 A3C depletion impairs the NF‐κB signaling pathway

**Fig. 2:**
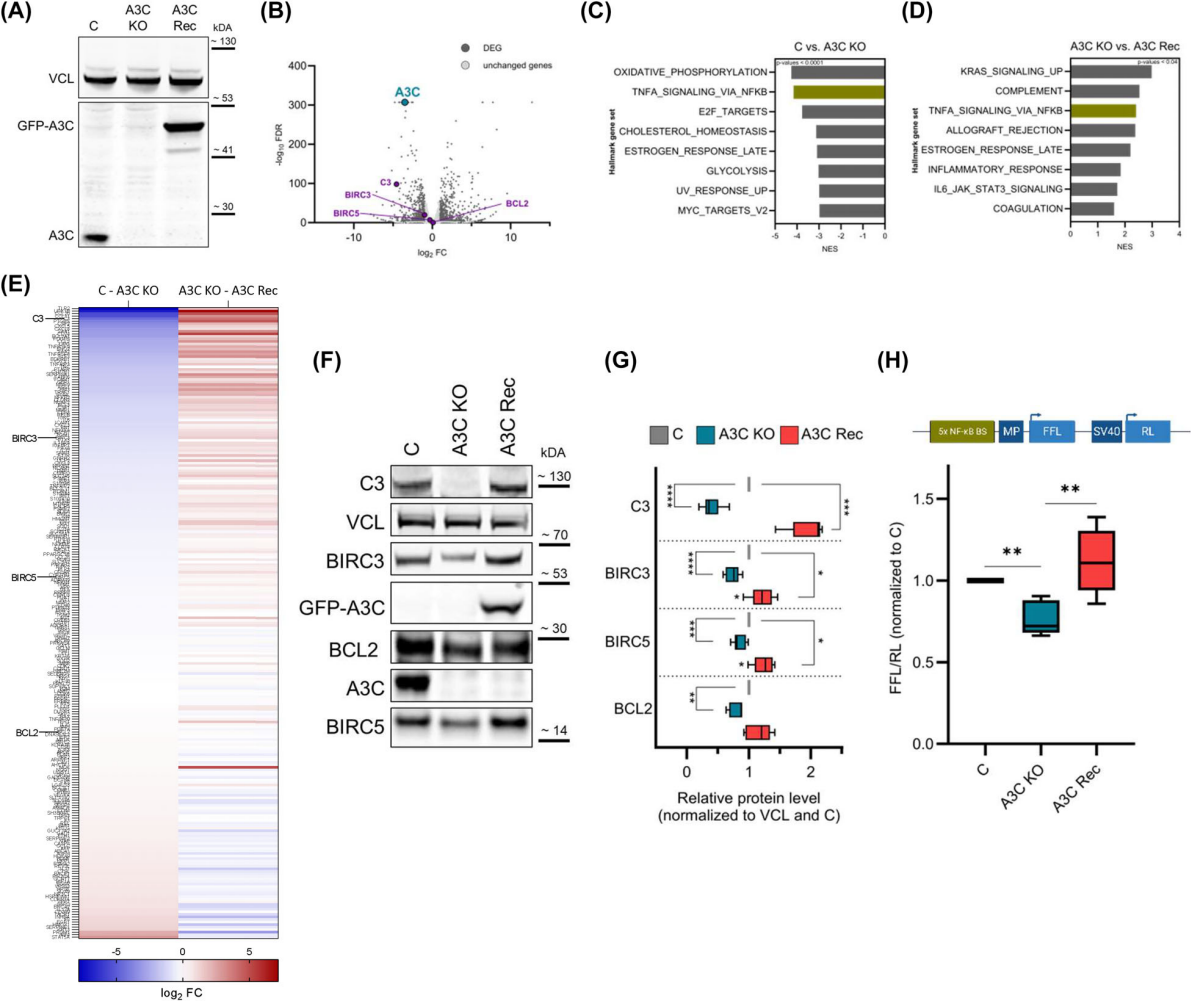
A3C depletion in 786‐O impairs NF‐κB signaling. (A) Western blot (WB) indicates the A3C levels in 786‐O control (C), CRISPR‐Cas9‐mediated A3C knockout (A3C KO) and A3C KO cells with re‐inserted GFP‐A3C (A3C Rec) cells (*n* = 3). VCL was used as the loading control. (B) Volcano plot of log_2_ mRNA fold changes (FC) plotted against the −log_10_ FDR (false discovery rate) shows differentially expressed genes (DEG; dark gray dots; FC > 2 or FC < −2) in 786‐O C and A3C KO cells. Exemplary NF‐κB target genes are highlighted in purple. For all used conditions (C, KO, Rec) three replicates were subjected to RNA‐seq analyses. (C, D) Gene set enrichment analyses (GSEA) were performed using differentially expressed mRNAs in 786‐O C vs. A3C KO (C; *n* = 5864 genes) or A3C KO vs. A3C Rec (D; *n* = 2379 genes) and the HALLMARK gene sets (*n* = 50 gene sets; NES, normalized enrichment score). (E) Heat map indicates the log_2_ FC for NF‐κB target genes according to https://bioinfo.lifl.fr/NF‐KB / and www.bu.edu/nf‐kb/gene‐resources/target‐genes/ upon KO of A3C and rescue of A3C in 786‐O. (F) Protein levels of four NF‐κB target genes (C3, BIRC3, BIRC5, and BCL2) in 786‐O A3C KO and A3C Rec are depicted in a representative WB (*n* = 3). Vinculin (VCL) was used as the loading control. (G) The NF‐κB target genes C3, BIRC3, BIRC5, and BCL2 protein levels were quantified by normalization to VCL and 786‐O C (*n* = 3). (H) The Firefly luciferase (FFL) activity was determined in 786‐O C, A3C KO and A3C Rec cells (*n* = 5). As shown in the upper panel, the luciferase reporter contains besides a minimal promoter (MP) five NF‐κB binding sites (BS) in the promoter region of the FFL. The Renilla luciferase (RL) was used for normalization. *P < 0.05; **P < 0.01; ***P < 0.001; ****P < 0.0001 by unpaired, two‐tailed Student's *t* test (G, H). Data are representative of three (G) or five (H) independent experiments (5–95 percentile in G and H).

Aiming to evaluate a disease promoting role of A3C, we generated A3C knockout (KO) cells using the CRISPR/Cas9 genome engineering in 786‐O cells expressing intermediate levels of A3C (Fig. 2A). In A3C‐KO cells, we further re‐introduced GFP‐A3C for recovery studies (A3C Rec; Fig. 2A). How A3C deregulation affects gene expression was evaluated by RNA sequencing (RNA‐seq). This revealed global gene expression profiles upon A3C deletion, resulting in 645 significantly down‐ and 656 significantly upregulated transcripts (up: FC ≥ 2, down: FC ≤ −2; FDR ≤ 0.01 was considered significant; Fig. 2B). Gene expression profiling further indicated the significant downregulation of 340 genes in A3C KO cells, which were substantially elevated by restoring A3C expression (FDR ≤ 0.01; data not shown). To assess this directly, we compared transcript levels in A3C‐KO cells with A3C rec cells (No FC restriction, FDR < 0.001, Fig. S2A,B). This demonstrated that A3C re‐expression restored the abundance of ~ 60% of genes downregulated by A3C‐KO. On a side note, we noticed a strong downregulation of A3C mRNA itself in A3C KO cell populations. This is frequently observed for CRISPR/Cas9‐mediated KO cells, indicating a degradation of A3C mRNA by nonsense‐mediated decay (NMD) [65]. Gene set enrichment analyses (GSEA) in the cancer Hallmark gene set collection, using the fold change of gene expression observed in C vs. A3C KO and A3C KO vs. A3C Rec for ranking, demonstrated consistent stimulation by A3C for the “TNFA SIGNALING VIA NFκB” gene set (Fig. 2C,D and Fig. S3A,B). In‐depth analyses of the RNA‐seq data revealed that a substantial fraction of NF‐κB inducible genes (~ 25%; *P* < 0.01; RPKM > 0.1) that are altered in A3C KO cells are recovered upon A3C re‐expression (Fig. 2E). In addition, the transcript levels of various chemokines and cytokines were altered upon A3C modulation (Fig. S2C). This included well known activators of the NF‐κB pathway like TGF‐β and IL18 [66, 67, 68]. Further investigation of four NF‐κB target genes (C3, BIRC3, BIRC5, and BCL2) by western blot analyses confirmed aberrant expression in 786‐O A3C KO and re‐expression in A3C Rec cells (Fig. 2F,G). For further validation of these findings by complementary studies, we utilized an independent silencing mechanism (shRNAs) to deplete A3C in cell populations and encompassed another ccRCC‐derived cell line, 769‐P, to evaluate the conservation of A3C‐dependent regulation. In both cell models, the constitutive knockdown of A3C (shA3C) substantially reduced protein levels of the NF‐κB‐driven genes C3, BIRC3, BIRC5 and BCL2 (Fig. S3C,D). Investigation of additional NF‐κB inducible genes by quantitative reverse transcription PCR (RT‐qPCR) in 786‐O and 769‐P cells confirmed vastly decreased expression upon A3C depletion (e.g., CSF2, TNFRSF9, TNFAIP3, VEGFA, and BIRC4; Fig. S3E).

Aiming to evaluate if A3C influences NF‐κB‐dependent transcriptional activity, we explored the activity of a luciferase reporter comprising NF‐κB binding motifs in the minimal promoter region (Fig. 2H). Consistent with the broad, A3C‐dependent deregulation of NF‐κB‐regulated gene expression in non‐stimulated cells, reporter activity was decreased by A3C deletion and elevated by its re‐expression (Fig. 2H and Fig. S3F). Collectively, these results indicate that A3C is a regulator of basal NF‐κB activity in renal cancer cells.

Consistent with previous reports that determined elevated NF‐κB activity in ccRCC [69], we confirmed significantly upregulated expression of three members of the NF‐κB transcription factor family (NF‐κB2, RelA, and RelB) in our ccRCC cohort (Fig. S4A). Elevated expression of NF‐κB2 and RelB was moreover associated with reduced overall survival probability (Fig. S4B,C). Likewise, elevated expression of NF‐κB inducible and A3C‐regulated genes in our ccRCC cohort, such as C3, BIRC3 and BIRC5 (Fig. S4E), was associated with decreased overall survival probability (Fig. S4F–H). These findings suggest that A3C contributes to adverse disease outcome in ccRCC by enhancing the expression of oncogenic factors like BIRC5 (survivin) in a potentially NF‐κB dependent manner.

### 3.3 RNA co‐immunoprecipitation identifies NF‐κB signaling regulators as mRNA targets of A3C

**Fig. 3:**
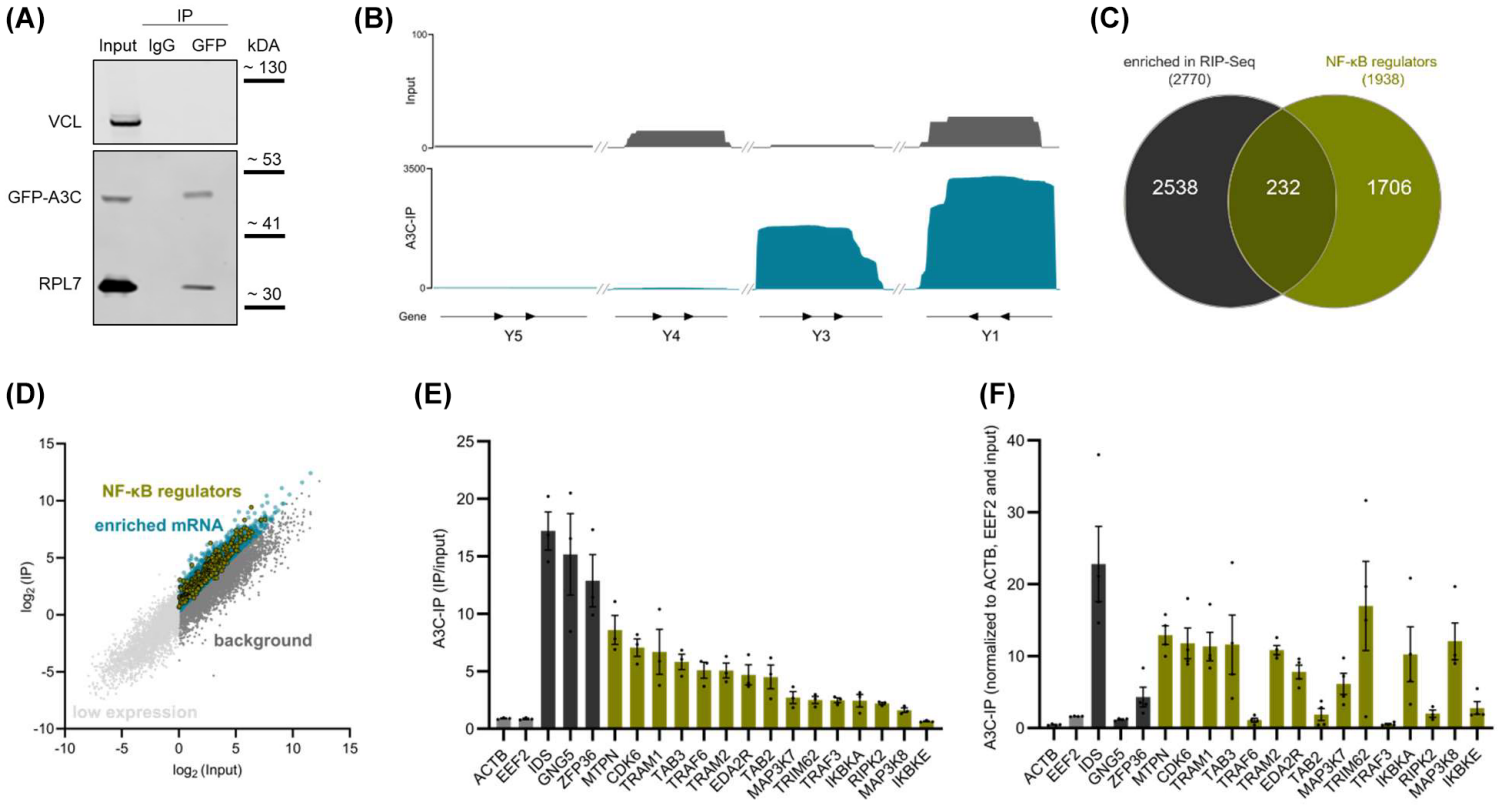
A3C associates with NF‐κB signaling pathway regulator mRNAs. (A) 786‐O A3C recovery (Rec) cells were used for immunoprecipitation (IP) with an anti‐GFP antibody and subjected to western blot analyses (*n* = 3). Ribosomal protein L7 (RPL7) and vinculin (VCL) served as positive and negative controls, respectively. Beads incubated with a non‐targeting antibody (IgG) were used as specificity control. (B) Inputs and normalized RNA co‐immunoprecipitation (RIP)‐seq reads of A3C‐IPs (*n* = 3) were investigated at four Y RNA loci to assess the reported association of A3C with Y RNAs. (C) The venn diagram shows the overlap of RIP‐seq enriched transcripts (FC (fold change) ≥ 2; *P* < 0.05; RPKM (reads per kilobase of transcript per million mapped reads) in input > 0.1) with reported NF‐κB signaling regulators (gene list obtained from www.gsea‐msigdb.org/gsea/msigdb/). (D) Scatter plot shows results of the RIP‐seq in 786‐O A3C Rec cells. High‐confidence binding partners of A3C are marked in blue (A3C‐IP/input > 1). Within this group, NF‐κB signaling regulators are colored in yellow. Transcripts with a ratio of A3C‐IP/input ≤ 1 are considered as background (gray). Transcripts with low expression (average RPKM in input < 0.1) are depicted in light gray. (E) Bar plot presents examples of enriched transcripts detected in RIP‐seq of the A3C‐IPs (IDS, GNG5 and ZFP36 within top 30). Reported NF‐κB signaling pathway regulators are depicted in yellow. (F) Bar plot indicates the same transcripts as in (E) obtained in separate A3C‐IPs (*n* = 3) and analyzed with real time quantitative PCR (RT‐qPCR). Note that the majority of enriched NF‐κB signaling regulator transcripts identified in the RIP‐seq also shows enrichment in A3C‐IPs analyzed by RT‐qPCR (A3C‐IP/input > 1). Data are representative of three independent experiments (mean ± SEM in E and F).

To investigate the molecular mechanisms underlying A3C‐dependent regulation of gene synthesis and NF‐κB activity, we initially aimed at identifying A3C‐associated (m)RNAs by RIP (RNA immunopurification) from stable 786‐O A3C Rec cells. Western blot analysis confirmed that GFP‐A3C was efficiently purified with anti‐GFP‐coated magnetic beads (Fig. 3A). Since global A3C interaction studies are not available to date, we consulted the BioGRID database. Among A3C‐interacting proteins reported by BioGRID, we observed other canonical RNA‐binding proteins (e.g., ELAVL1 and HNRNPK) as well as ribosomal subunits (e.g., RPL7, RPL28, and RPS13). In western blot analyses, RPL7 served as a positive control, indicating the association of A3C with components of mRNPs.

A3 family proteins were described to promiscuously interact with diverse RNA classes, including non‐coding RNAs like 7SL RNA, Alu RNA, and Y RNAs as well as mRNAs [70, 71]. Consistent with the proposed promiscuous RNA‐association of A3 proteins, the investigation of RNAs co‐purified with A3C revealed more than > 5300 protein‐coding and non‐coding transcripts substantially enriched over controls (FC compared to input ≥ 2; average RPKM in input > 0.1; Table S3). In agreement with reported association of A3C with Y RNAs, RIP‐studies confirmed robust association of A3C with Y1 and Y3 RNAs (Fig. 3B). We also observed that a variety of potential A3C target RNAs belong to pathways not significantly affected by A3C modulation, which might imply a broad A3C target spectrum (Fig. S5). Subsequently, we compared all significantly enriched protein‐coding genes from the A3C‐IP (FC ≥ 2; *P* < 0.05; average RPKM in input > 0.1) with reported NF‐κB signaling pathway regulators (gene sets obtained from GSEA Molecular Signatures database). We identified 232 putative mRNA‐binding partners of A3C that are described to be linked to the NF‐κB signaling pathway (Fig. 3C,D, Table S3), suggesting that A3C could affect the NF‐κB signaling pathway by modulating its regulators.

To further evaluate if A3C may modulate NF‐κB activity by associating with mRNAs encoding regulators of NF‐κB signaling, the prime candidate target transcripts identified by RIP‐RNAseq were re‐evaluated by RT‐qPCR upon immunopurification from 786‐O A3C Rec cells (Fig. 3E,F). For the most part, mRNA‐association of A3C could be recapitulated by RT‐qPCR. Importantly, robust association was confirmed with key regulators of NF‐κB signaling, for example, CDK6, and IKBKA. These findings implied that A3C influences NF‐κB signaling by modulating the fate of some mRNAs encoding NF‐κB regulators via mRNPs.

A3C is a cytidine deaminase reported to edit the sequence of associated transcripts. To evaluate putative mutations as source of modified NF‐κB activity, we determined potential editing sites in the RNA‐seq data of 786‐O C and A3C KO cells. These analyses suggested several hundred C‐to‐U editing events, relying on disturbed A3C expression. Aiming to identify high‐confidence editing events, events were further filtered by read counts of the editing site and minor modification rates observed by A3C‐KO in comparison to controls. This revealed 84 high‐confidence target mRNA for A3C‐dependent C‐to‐U editing (Table S1). In‐depth analyses demonstrated that most of these editing sites are either not recovered by A3C re‐expression, are located in the 3′ untranslated region (3′ UTR) or at the wobble position of a codon causing no amino acid alteration in the final protein. Finally, we determined that putative editing occurs in seven transcripts that are also potential binding partners of A3C according to RIP‐seq studies. Interestingly, none of these editing candidates are linked to the NF‐κB signaling pathway. Overall, these findings indicate that A3C likely modulates NF‐κB activity in a largely deaminase independent function.

### 3.4 A3C depletion impairs the NF‐κB signaling pathway by restraining NF‐κB subunits in the cytoplasm

**Fig. 4:**
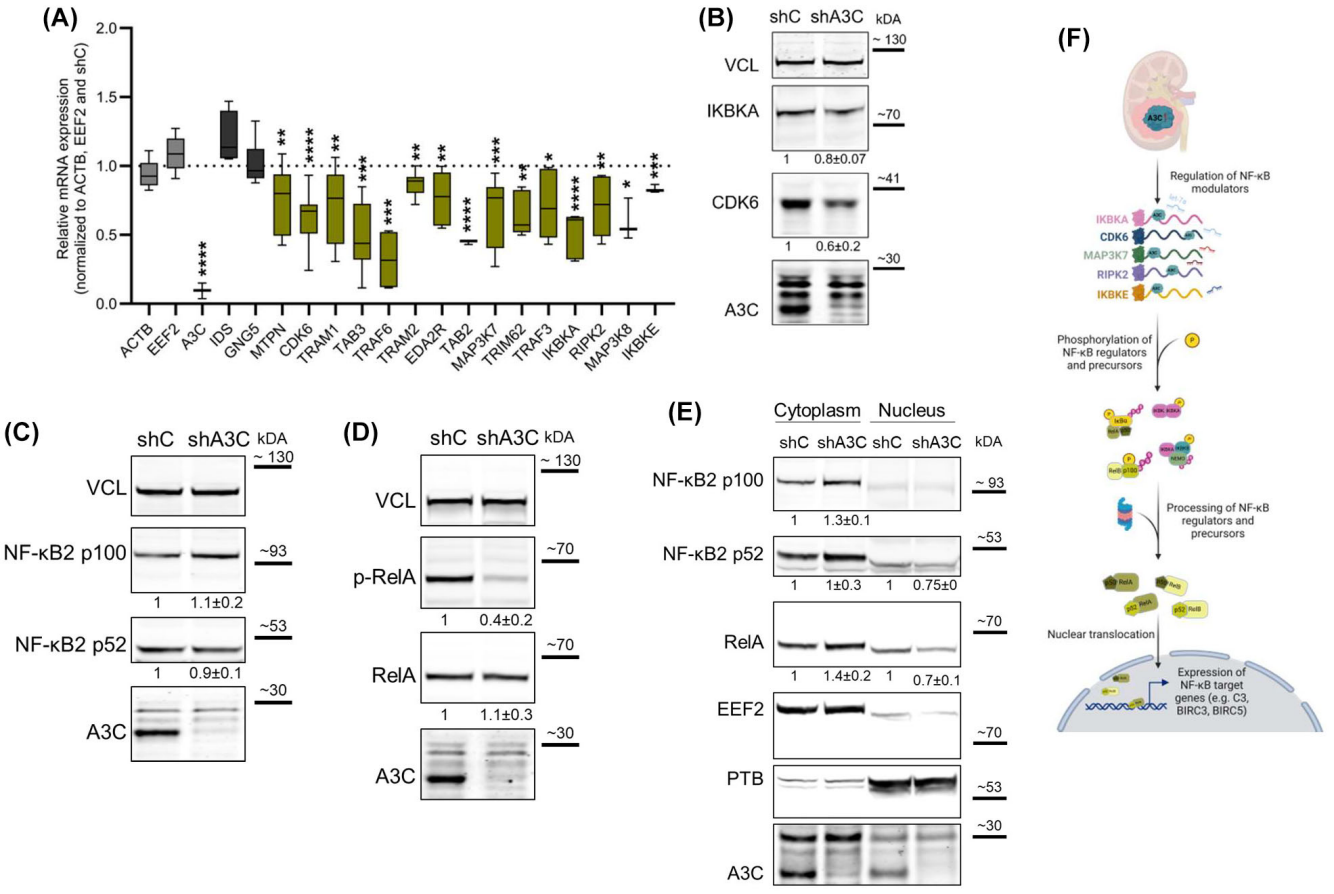
A3C depletion results in reduced expression of NF‐κB signaling pathway regulators and impaired nuclear translocation of NF‐κB subunits. (A) Transcript levels of NF‐κB signaling pathway regulators (marked in yellow) were analyzed upon stable A3C knockdown (shA3C) in 786‐O by real time quantitative PCR (RT‐qPCR, *n* = 3). Beta actin (ACTB) and eukaryotic elongation factor 2 (EEF2, light gray) served as negative controls. IDS and GNG5 (dark gray) are putative binding partners of A3C, but not considered NF‐κB signaling pathway regulators. (B) Western blot (WB) analyses confirmed decreased expression of CDK6 and IKBKA in 786‐O shA3C cells (*n* = 5). (C) WB indicates protein levels of the unprocessed (p100) and processed (p52) forms of NF‐κB2 in 786‐O shA3C cells (*n* = 6). (D) Phosphorylation status at Ser536 and total protein levels of RelA in 786‐O shA3C cells were characterized by WB analyses (*n* = 3). The phosphorylation signal was normalized to total RelA protein level. (E) Subcellular fractionation was performed using 786‐O shC and shA3C cells (*n* = 3). The distribution of the NF‐κB subunits NF‐κB2 and RelA among the cytoplasmic and nuclear fractions is depicted in the WB. To verify the subcellular fractionation process, EEF2 and PTB were used as positive controls for the cytoplasmic and nuclear fraction, respectively. Note that due to the usage of different buffers in the cytoplasmic and nuclear protein pool, we observed slight differences in the running behavior of the proteins. (F) The schematic depicts a putative regulatory mechanism of the NF‐κB signaling pathway by A3C. *P < 0.05; **P < 0.01; ***P < 0.001; ****P < 0.0001 by unpaired, two‐tailed Student's *t* test compared to 786‐O shC (A). Data are representative of three independent experiments (5–95 percentile in A). Protein levels were normalized to vinculin (VCL) and 786‐O control cells (shC) in five (B), six (C) or three (D, E) biological replicates; mean ± SD is indicated below the representative WB (B–E).

To test how A3C modulates the fate of candidate target mRNAs identified by RIP‐analyses, we next assessed their abundance upon constitutive depletion of A3C in 786‐O and 769‐P cells. Strikingly, we observed a robust and broad decrease of candidate target mRNAs encoding NF‐κB regulators in both cell models by A3C depletion (Fig. 4A and Fig. S6A). In contrast, control (ACTB and EEF2) as well as mRNAs not encoding NF‐κB regulators (IDS and GNG5 mRNAs) remained largely unchanged. For two selected targets, CDK6 and IKBKA, downregulation was confirmed at the protein level (Fig. 4B and Fig. S6B). This indicated that A3C‐associated transcripts encoding NF‐κB regulators tend to result in decreased mRNA abundance and protein synthesis.

NF‐κB activity is frequently deregulated in RCC [18]. Specifically, RelA‐containing complexes appear to be the dominant form of NF‐κB in ccRCC‐derived cell lines [69]. Additionally, these complexes are often re‐located to the nucleus [69]. In our further analyses, we also included NF‐κB2, as it was highly upregulated in our small ccRCC cohort (Fig. S4A), covering subunits of both the canonical and non‐canonical NF‐κB pathway. Subsequently, we determined the protein levels of NF‐κB2 and RelA upon A3C depletion. Total protein levels of NF‐κB2 and RelA remained unchanged in 786‐O (Fig. 4C,D) and 769‐P cells (Fig. S6C). Importantly, however, we noticed a stark (~ 2‐fold) reduction in S536‐phosphorlyation of RelA by constitutive A3C knockdown (Fig. 4D). S536 of RelA is phosphorylated by various kinases leading to the nuclear translocation, accumulation, and enhancement of RelA‐driven transcription [72, 73]. How altered phosphorylation of RelA upon A3C knockdown influences its subcellular distribution was analyzed by subcellular fractionation in 786‐O and 769‐P cells (Fig. 4E and Fig. S6D,E). Successful fractionation was validated by enrichment of EEF2 in the cytoplasmic and PTB in the nuclear fraction, respectively. Consistent with disturbed subcellular sorting and transcriptional activity in the NF‐κB pathway, A3C depletion was associated with a 30–40% reduction of processed NF‐κB2 p52 and RelA levels in the nucleus, whereas their abundance increased by approximately 40% in the cytoplasm. The results collectively suggest that A3C plays a role in modulating the expression of NF‐κB signaling pathway activators, possibly by binding and stabilizing the respective transcripts and facilitating the efficient nuclear translocation of NF‐κB2 p52 and RelA, which likely influences NF‐κB‐driven transcription as schematically depicted in Fig. 4F.

### 3.5 A3C is a stress responsive survival factor for tumor growth *in vivo*

**Fig. 5:**
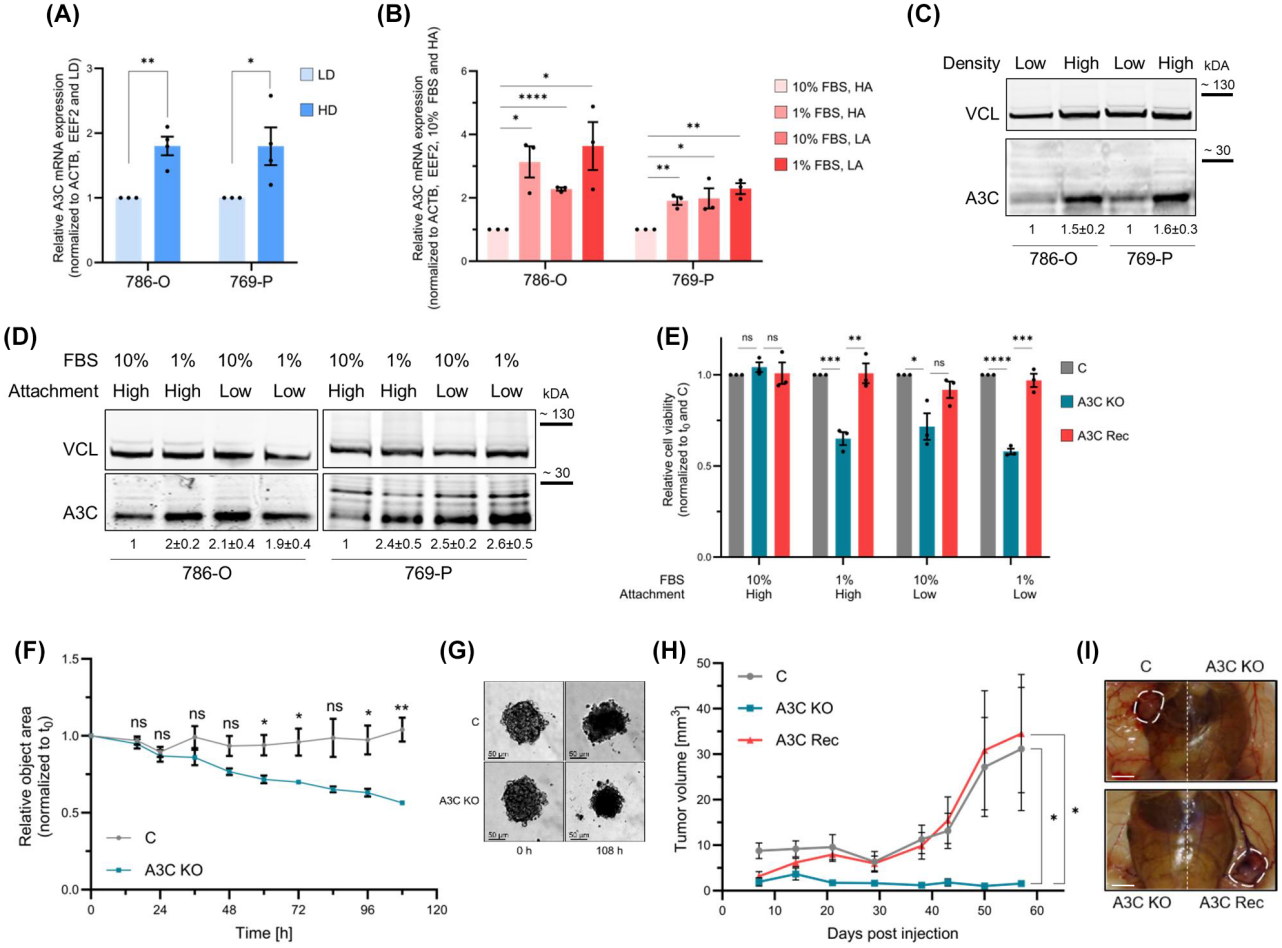
Elevated A3C expression is associated with cell survival *in cellulo* and *in vivo*. (A–D) Expression levels of A3C were investigated in 786‐O and 769‐P wild‐type (WT) cells under diverse growth conditions. (A) Bar plots indicate mRNA levels of A3C under low (LD) or high (HD) density growth conditions (*n* = 4). (B) Cells were cultured under fetal bovine serum (FBS) depleted conditions (1% FBS) combined with low (LA) or high (HA) attachment. A3C mRNA levels were determined (*n* = 3). (C, D) Western blot (WB) analyses demonstrate A3C protein levels under LD compared to HD conditions (C; *n* = 4) and FBS‐depleted conditions combined with LA or HA (D; *n* = 3). (E) Anoikis growth conditions and FBS depletion were applied to 786‐O control (C), A3C knockout (KO) and A3C recovery (Rec) cells (*n* = 3). Cell survival was investigated after 5 days. (F, G) 3D spheroids of 786‐O C and A3C KO cells were monitored for 5 days. The object area of the spheroids was quantified and normalized to the initial size (F; *n* = 3). Representative images of cell spheroids at day 0 and day 5. Scale bar represents 50 μm (G). (H, I) Foxn1^nu/nu^ mice were subcutaneously injected with 786‐O C, A3C KO, and A3C Rec cells (*n* = 8 mice per group). Tumor growth was monitored for several weeks (H). Representative images of final tumors in nude mice are shown. Scale bar indicates 1 cm (I). *P < 0.05; **P < 0.01; ***P < 0.001; ****P < 0.0001 by unpaired, two‐tailed Student's *t* test (A, B, E, F, H). Data are representative of four (A) or three (B, E, F) independent experiments (mean ± SEM in A, B, E, F, H). Protein levels were normalized to vinculin (VCL) and control growth conditions (LD for C; 10% FBS and HA for D) in four (C) or three (D) biological replicates; mean ± SD is indicated below the representative WB (C, D).

Aiming to evaluate if A3C serves essential roles in modulating the oncogenicity of RCC tumor cells, we evaluated phenotypic consequences of deregulated A3C expression in ccRCC models. To this end, we initially examined A3C expression in parental 786‐O and 769‐P cell populations under various cellular stress conditions, which are common in progressing neoplasia and were reported to activate NF‐κB signaling [74, 75]. Intriguingly, A3C expression was enhanced with elevated tumor cell density (HD, high density), culturing at low‐attachment conditions (LA, low attachment), and serum (FBS, 1 vs. 10%) deprivation (Fig. 5A–D). In view of the reported activation of the NF‐κB pathway by aforementioned stress stimuli and anti‐cancer drug treatments [76], these findings suggested a role of A3C in fostering NF‐κB‐dependent stress responses in ccRCC.

This was tested directly by exposing 786‐O A3C KO and A3C Rec cells to adhesion and starvation stress. A3C KO cells exhibited significantly increased susceptibility to the respective stress conditions, as evidenced by impaired cell viability compared to controls (Fig. 5E). Cell viability was significantly restored by A3C re‐expression, indicating a pivotal role of A3C in sustaining tumor cell vitality and resilience under unfavorable conditions. In agreement, reduced stress resilience was also observed by constitutive depletion of A3C in 786‐O and 769‐P cells (Fig. S7A,D). Notably, A3C modulation only impacted stressed cells, since barely any change in cell viability was observed under optimal growth conditions (HA, 10% FBS). If stress tolerance is indeed modulated by NF‐κB signaling in the chosen cell models was explored by depleting NF‐κB2 and RelB in 769‐P exposed to the same conditions as cells perturbed in A3C expression (Fig. S7G,H). Both these depletions robustly, in the case of NF‐κB2 pronounced, increased susceptibility to adhesion and mitogen stress. This suggested a phenocopy of perturbing NF‐κB signaling directly and perturbing A3C expression.

If A3C also impacts on tumor growth was initially accessed in 3D spheroid growth studies (Fig. 5F,G). In both ccRCC cell models, 786‐O and 769‐P, the depletion of A3C significantly impaired spheroid growth, which became evident already 60 h after spheroid seeding (Fig. 5F,G and Fig. S7B,C,E,F). These findings prompted us to test if A3C also impacts tumor growth *in vivo*, using subcutaneous (s.c.) 786‐O xenograft models (Fig. 5H,I). The monitoring of tumor volume over time revealed that the deletion of A3C (A3C KO, blue) essentially abolished tumor growth. In sharp contrast, the re‐expression of A3C fully restored tumor growth in comparison to parental cell populations. These findings provide compelling evidence that A3C is a strong enhancer of RCC tumor growth.

## 4 Discussion

In this study, we for the first time demonstrate that elevated A3C expression is associated with increased NF‐κB activity in ccRCC‐derived cells, elevated stress resilience of tumor cells *in vitro* and substantially fostered tumor growth *in vivo*. Our studies provide strong evidence that this oncogenic role of A3C is intimately linked to A3C‐dependent stimulation of NF‐κB signaling but largely independent of the cytidine deamination of A3C. These findings are largely consistent with observations suggesting that A3D and A3G, close homologs of A3C, are unfavorable prognostic markers in ccRCC [42, 43, 77], but their role remained largely elusive. A tumorigenic role of APOBEC proteins only been demonstrated for A3A and A3B, which were implicated in mutational burden associated with increased tumor cell proliferation, survival, and drug resistance in various cancers [78, 79, 80, 81, 82, 83, 84].

Our studies imply that A3C promotes tumorigenicity of ccRCC tumor cells in an at least partially NF‐κB‐dependent manner. Activation of the NF‐κB transcription factors encompasses a diverse array of stimuli, a variety of receptors and a complex regulatory network (reviewed in Refs [30, 33, 34, 85]). Importantly, NF‐κB regulation essentially relies on signal‐induced proteolytic degradation of NF‐κB inhibitors (IκBs), referred to as the canonical or classical pathway [17, 86, 87]. An additional non‐canonical or alternative pathway introduces other regulatory mechanisms, for example, post‐translational modifications like phosphorylation, which among other effects especially modulate nuclear‐cytoplasmic trafficking of transcriptional regulators (reviewed in Refs [32, 88, 89, 90]). We demonstrate that A3C is involved in this regulation by demonstrating that A3C depletion/deletion results in both, altered phosphorylation of RelA and the nuclear translocation of RelA as well as NF‐κB2. Notably, this A3C‐depedent control affects the overall activity of NF‐κB luciferase reporters and expression of various NF‐κB target genes in RCC tumor cells. These findings imply a role of A3C in enhancing NF‐κB activity by modulating the activation of transcriptional regulators of the NF‐κB pathways. This activity is obviously linked to the A3C‐dependent regulation of factors with reported roles in NF‐κB signaling like protein kinases (e.g., CDK6, MAP3K7, IKBKA, and RIPK2), signaling receptors (e.g., EDA2R), as well as signaling adaptors (e.g., TAB3 and TRAF6). Our RIP‐seq analyses indicate association of A3C with the respective mRNAs without stark evidence for C‐to‐U editing. However, most of A3C‐associated mRNAs encoding NF‐κB regulators were decreased by A3C depletion and deletion, suggesting that A3C controls NF‐κB activity by modulating the expression of factors controlling activation of gene synthesis within the canonical and non‐canonical NF‐κB pathways.

Most studies on the APOBEC3 protein family in cancer focus on cytidine deamination activity and mutational burden. However, members of the family also have been implicated in modulating mRNA fate in a deaminase independent manner. For instance, A3G counteracts miRNA‐directed gene repression independent of its cytidine deaminase activity [91]. This has also been described for other APOBEC3 family members and was reported to impact on regulation by various miRNAs, for example, miR‐10b, miR‐16, miR‐25, miR‐29, and let‐7a [92, 93, 94]. Although the underlying mechanism remains unclear to date, it has been proposed that A3G prevents the decay of miRNA‐targeted mRNAs in processing bodies (P‐bodies) and instead promotes the association of mRNA with polysomes [93, 95]. Interestingly, A3G is located in P‐bodies and stress granules [96, 97]. In accordance with other reports [56, 98], we also confirmed the co‐localization of A3C with AGO2 and YB1 upon applying arsenate stress to 786‐O A3C Rec cells, indicating the localization of A3C in P‐bodies and stress granules, respectively (data not shown). Thus, the function of A3C might be connected with P‐body‐related miRNA‐mediated mRNA repression. In ccRCC, an inverse correlation was identified between immune‐associated genes and the expression of multiple miRNAs, such as miR‐149 and miR‐508, suggesting miRNAs to be key regulators of dysregulated expression of genes connected to immune responses [99]. Furthermore, members of the let‐7 family, considered tumor‐suppressive miRNAs, are downregulated in many cancers including ccRCC [100, 101, 102, 103]. Interestingly, several positive regulators of the NF‐κB pathway, identified as RNA binding partners of A3C such as CDK6, IKBKA, MAP3K7, MTPN, and TAB3, harbor let‐7 binding sites in their mRNAs. Additionally, these putative binding partners exhibit reduced expression levels upon depletion of A3C (Fig. 3E,F). Collectively, these findings emphasize that A3C might also control the expression of NF‐κB regulators by antagonizing their miRNA‐dependent downregulation.

As previously reported, CDK6, in addition to other kinases like IKBK, IKBKB [104], or TBK1 [105, 106], was identified as a novel kinase phosphorylating RelA at Ser536 [107]. Accordingly, increased CDK6 activity would promote phosphorylation at this site, leading to the translocation of RelA into the nucleus and the subsequent induction of NF‐κB target gene transcription. Upon A3C depletion, we observed reduced nuclear translocation of RelA and NF‐κB2 p52, along with an accumulation of unprocessed subunits in the cytoplasm (Fig. 4E and Fig. S6E). This was associated with the downregulation of several NF‐κB target genes such as BIRC3, BIRC5 and BCL2 (Fig. 2E–G and Fig. S3C–E). These NF‐κB target genes are important for cell survival, as they primarily impair apoptosis [108, 109, 110]. In ccRCC, inhibitor of apoptosis (IAP) proteins like BIRC3 and BIRC5 have been previously correlated with advanced stages and more aggressive ccRCCs, designating them as unfavorable prognostic markers (Fig. S4) [111, 112, 113].

We demonstrate that A3C is dispensable for tumor cell growth under favorable conditions. This is concise with the finding that the single Apobec3 gene encoded in the mouse genome is neither essential for murine development, survival, nor fertility [114]. However, our studies also reveal that A3C fosters stress resilience of RCC‐derived cells *in vitro* and is essential for tumor growth *in vivo*. Notably, A3C expression is enhanced in ccRCC, characterized by VHL inactivation, a strong degrader of APOBEC3 family proteins [115]. Collectively, this suggests that the upregulation of A3C in ccRCC promotes tumor growth and disease progression by increasing stress‐resilience in a potentially NF‐κB‐dependent manner.

Consequently, inhibition of A3C could be a novel strategy for developing treatment options in ccRCCs. Indeed, ongoing studies focus on the use of nucleic acid‐based inhibitors [116, 117], substrate‐mimetics [118], or small molecule inhibitors [119] to counteract APOBEC3 activity. In addition, we conducted initial investigations to assess the impact of A3C on the efficacy of compounds used in the treatment of advanced ccRCC. Sorafenib, Pazopanib, and Sunitinib, all known inhibitors of multiple tyrosine protein kinases and receptor tyrosine kinases, have been reported to reduce tumor vascularization, trigger cancer cells and, thus, promote tumor shrinkage [120, 121, 122]. After determining the EC_50_ values for each drug, cell viability was assessed in 786‐O A3C KO cells upon drug treatment. Cells with depleted levels of A3C exhibited significantly reduced viability when treated with Pazopanib or Sunitinib (Fig. S7I), indicating that ccRCC‐derived cells may become more susceptible to tyrosine kinase inhibitor treatment upon A3C depletion or inhibition. These findings suggest benefits of targeted A3C‐inhibition in combination with established treatment regimens. Further research in this area is essential to explore the potential benefits of this combination therapy.

## 5 Conclusion

This study demonstrates that the RNA‐binding protein APOBEC3C (A3C) is frequently upregulated in clear cell renal cell carcinoma (ccRCC). We highlight that A3C represents a stress‐responsive factor that is crucial for ccRCC cell survival especially under adverse growth conditions. On the mechanistic level, we determined by loss of function and recovery studies that A3C levels are largely congruent with NF‐κB activity, a pro‐survival pathway in ccRCC. Hence, we also identified that A3C binds and potentially stabilizes mRNAs encoding positive regulators of the NF‐κB pathway. This in turn leads to enhanced translocation of subunits of the NF‐κB family and increased NF‐κB target gene expression. In summary, this study emphasizes the unappreciated role of A3C in promoting ccRCC tumor growth, underlining its potential as target for future therapeutic avenues.

## Conflict of interest

The authors declare no conflict of interest.

## Author contributions

NH and MK conceptualized the study and performed experiments. MK executed the mouse experiments. HT, SH, and MG provided resources, helped with interpretation of the data and reviewed it critically for important content. NH, DM, and MK conducted RNA sequence analyses and the statistical analyses. NH wrote the original draft of the manuscript. All authors reviewed and edited the manuscript.

## Supporting information


**Fig. S1.** Expression of A3 family members in a separate, small RCC cohort.
**Fig. S2.** RNA‐seq in 786‐O CRISPR/Cas9‐mediated A3C KO and A3C Rec cells.
**Fig. S3.** Confirmation of the impaired NF‐κB signaling pathway upon stable A3C knockdown.
**Fig. S4.** Clinical relevance of NF‐κB family members and NF‐κB target genes in RCC.
**Fig. S5.** Binding partners of A3C belong to diverse HALLMARK gene sets.
**Fig. S6.** A3C depletion in 769‐P results in reduced expression of NF‐κB signaling pathway regulators and impaired nuclear translocation of NF‐κB subunits.
**Fig. S7.** A3C regulates cell viability under diverse growth conditions and drug treatment.

**Table S1.** Putative A3C editing sites as assessed by RNA sequencing (A3C KO vs Control and Recovery populations).

**Table S2.** Materials used in this study.

**Table S3.** Analysis of A3C‐interacting RNAs by RIP‐Seq.

## Data Availability

The data generated in this study are available within the article and its Supplement Figures S1 files. Global RNA expression analyses (RNA‐seq) are available upon request from the corresponding author.
