# Supplementary material for: APOBEC3C‐mediated NF‐κB activation enhances clear cell renal cell carcinoma progression
Source: Mol Oncol. 2024 Aug 26;19(1):114–32. doi: 10.1002/1878-0261.13721 (PMC11705732; doi:10.1002/1878-0261.13721)
Supplement: Supplementary file 1 — Fig. S1. Expression of A3 family members in a separate, small RCC cohort. Fig. S2. RNA‐seq in 786‐O CRISPR/Cas9‐mediated A3C KO and A3C Rec cells. Fig. S3. Confirmation of the impaired NF‐κB signaling pathway upon stable A3C knockdown. Fig. S4. Clinical relevance of NF‐κB family members and NF‐κB target genes in RCC. Fig. S5. Binding partners of A3C belong to diverse HALLMARK gene sets. Fig. S6. A3C depletion in 769‐P results in reduced expression of NF‐κB signaling pathway regulators and impaired nuclear translocation of NF‐κB subunits. Fig. S7. A3C regulates cell viability under diverse growth conditions and drug treatment. [file MOL2-19-114-s003.zip › Supplemental_Figure_Legends.docx]

#### **Supplemental Figure Legends**

**Supplemental Figure 1. Expression of A3 family members in a separate, small RCC cohort.** (**A**) The volcano plot shows upregulated (log_2_ FC ≥ 1) and downregulated (log_2_ FC ≤ -1) genes in RNA-seq data from ccRCC patients. FDR ≤ 0.1 was considered significantly changed. (**B**) A3C expression was analyzed in diverse RCC subtypes: clear cell RCC (ccRCC), papillary RCC (papRCC) type 1 (T1) or type 2 (T2) and chromophobe RCC (chrRCC). **P < 0.01; ***P < 0.001 by unpaired, two-tailed Student’s t test.

**Supplemental Figure 2. RNA-seq in 786-O CRISPR/Cas9-mediated A3C KO and A3C Rec cells.** (**A**) Heat map shows log_2_ FC of significantly differentially expressed genes in 786-O C, A3C KO and A3C Rec cells. All three replicates per condition are indicated. (**B**) The upper Venn diagram shows the overlap of downregulated genes in A3C KO compared to C and upregulated genes in A3C Rec compared to A3C KO (FPKM in C > 0.1; FDR < 0.001). The lower Venn diagram shows the overlap of upregulated genes in A3C KO compared to C and downregulated genes in A3C Rec compared to A3C KO (FPKM in C > 0.1; FDR < 0.001). (**C**) Heat map indicates the log_2_ FC of chemokines and cytokines according to [**www.bu.edu/nf-kb/gene-resources/target-genes/**](http://www.bu.edu/nf-kb/gene-resources/target-genes/) upon KO of A3C and rescue of A3C in 786-O (FPKM in C > 0.005) [Turner MD, Nedjai B, Hurst T, et al. (2014). Cytokines and chemokines: At the crossroads of cell signalling and inflammatory disease. Biochim Biophys Acta 1843, 2563-2582].

**Supplemental Figure 3. Confirmation of the impaired NF-κB signaling pathway upon stable A3C knockdown.** (**A** and **B**) Enrichment plots of the HALLMARK gene set ‘TNFA SIGNALING VIA NFκB‘ from GSEA using DEG in sgC vs. sgA3C (**A**; NES=-4.16; q < 0.0001) and sgA3C vs. sgA3C Rec (**B**; NES=2.41; q = 0.002) are presented. (**C**, **D** and **E**) Decreased levels of selected NF-κB target genes are quantified in 786-O and 769-P upon stable knockdown of A3C (shA3C). (**C**) Representative WB displays reduced protein levels of C3, BIRC3, BIRC5 and BCL2. (**D**) Protein levels were quantified by normalization to VCL and shC (n≥3). (**E**) NF-κB target genes were investigated at mRNA level (n=3). Note that the majority of the randomly selected NF-κB target genes shows lower expression upon stable knockdown of A3C in 786-O and 769-P. (**F**) The box and whiskers plot indicates reduced NF-κB guided FFL activity in 786-O and 769-P upon stable knockdown of A3C (n=4). *P < 0.05; **P < 0.01; ***P < 0.001; ****P < 0.0001 by unpaired, two-tailed Student’s t test compared to shC (**D**, **E** and **F**). Data are representative of at least three independent experiments (5-95 percentile in **D** and **F;** mean ±SEM in **E**).

**Supplemental Figure 4. Clinical relevance of NF-κB family members and NF-κB target genes in RCC.** (**A**) Relative mRNA expression of the NF-κB family members in ccRCC (n=8) and corresponding NT (n=8) is shown. (**B**, **C** and **D**) Overall survival of RCC patients based on different NF-κB2 (**B**), RelB (**C**) and RelA (**D**) expression status was investigated by Kaplan-Meier analyses. (**E**) Violin plot indicates relative mRNA expression of selected NF-κB target genes in ccRCC patients (n=8) and corresponding NT (n=8). (**F**, **G**, **H** and **I**) Overall survival of RCC patients based on different C3 (**F**), BIRC3 (**G**), BIRC5 (**H**) and BCL2 (**I**) expression status was examined by Kaplan-Meier analyses. **P < 0.01 by unpaired, two-tailed Student’s t test (**A** and **E**). Kaplan-Meier plots were generated using <http://gepia2.cancer-pku.cn/> with data obtained from TCGA (**B**-**D** and **F**-**I**).

**Supplemental Figure 5. Binding partners of A3C belong to diverse HALLMARK gene sets.** Scatter plots show putative binding partners of A3C identified in the RIP-seq of 786-O A3C Rec cells (mean FPKM in input > 0.1). Depicted are transcripts belonging to the HALLMARK gene set ‚TNFA_SIGNALING_VIA_NFKB‘ (yellow) in comparison to other HALLMARK gene sets that are not negatively enriched in 786-O C vs. A3C KO GSEA: ‚HEME_METABOLISM‘ (purple; **A**), ‚MITOTIC_SPINDLE‘ (blue; **B**) and ‚SPERMATOGENESIS‘ (black; **C**).

**Supplemental Figure 6. A3C depletion in 769-P results in reduced expression of NF-κB signaling pathway regulators and impaired nuclear translocation of NF-κB subunits.** (**A**) mRNA expression levels of NF-κB signaling pathway regulators (marked in yellow) were analyzed upon stable A3C knockdown in 769-P by RT-qPCR (n≥3). ACTB and EEF2 (light gray) were used as negative controls. IDS and GNG5 (dark gray) are putative binding partners of A3C, but not considered NF-κB signaling pathway regulators. (**B**) WB analyses confirmed decreased expression of CDK6 and IKBKA in 769-P shA3C cells (n=4). (**C**) Protein levels of the unprocessed (p100) and processed (p52) forms of NF-κB2 in 769-P shA3C cells are depicted in the WB (n=12). (**D** and **E**) Subcellular fractionation was performed using 769-P shC and shA3C cells. The distribution of the NF-κB subunits NF-κB2 (**D**; C, cytoplasm; N, nucleus; n=3) and RelA (**E**; n=2) among the cytoplasmic and nuclear fractions is shown in the WBs. EEF2 and PTB served as positive controls for the cytoplasmic and nuclear fraction, respectively. Note that due to the usage of different buffers in the cytoplasmic and nuclear fractions, we observed slight differences in the running behavior of the proteins. *P < 0.05; **P < 0.01; ***P < 0.001; ****P < 0.0001 by unpaired, two-tailed Student’s t test compared to 769-P shC (**A**). Data are representative of at least three independent experiments (5-95 percentile in **A**). Protein levels were normalized to VCL and 769-P control cells (shC) in at least three biological replicates (in **E**, two biological replicates; mean ±SD is indicated below a representative WB (**B**, **C**, **D** and **E**).

**Supplemental Figure 7. A3C regulates cell viability under diverse growth conditions and drug treatment.** (**A**) Cell survival under FBS depletion (1% FBS) combined with low (LA) or high attachment (HA) conditions was investigated in 786-O shA3C cells (n=3). (**B**) 3D spheroids of 786-O shC and shA3C cells were cultured for five days and the object area was quantified (n=6). (**C**) Representative images of the initial and terminal spheroids of 786-O shC and shA3C cells. (**D**-**F**) Experiments were performed as described in (**A**-**C**) with 769-P cells exhibiting stable A3C knockdown. (**G**) Cell viability of 769-P WT cells treated with diverse siRNAs was determined under various FBS and attachment conditions after 5 days (n=3). (**H**) Knockdowns of proteins targeted in (**G**) were detected by WB one day or five days after siRNA transfection. (**I**) 786-O C and A3C KO cells were treated with Sorafenib (3.5 µM), Pazopanib (15 µM) and Sunitinib (6 µM). Cell viability was determined after 48 h by CellTiterGlo (n=4). *P < 0.05; **P < 0.01; ***P < 0.001; ****P < 0.0001 by unpaired, two-tailed Student’s t test (**A**, **B**, **D** and **E**; compared to siC in **G**) and by Šídák's multiple comparisons test (**I**). Data are representative of three (**A**, **D** and **G**), six (**B** and **E**) or four (**I**) independent experiments (mean ±SEM in **A**, **B**, **D**, **E**, and **G**; 5-95 percentile in **I**).
