# Supplementary material for: APOBEC3C‐mediated NF‐κB activation enhances clear cell renal cell carcinoma progression
Source: Mol Oncol. 2024 Aug 26;19(1):114–32. doi: 10.1002/1878-0261.13721 (PMC11705732; doi:10.1002/1878-0261.13721)
Supplement: Supplementary file 1 — Fig. S1. Expression of A3 family members in a separate, small RCC cohort. Fig. S2. RNA‐seq in 786‐O CRISPR/Cas9‐mediated A3C KO and A3C Rec cells. Fig. S3. Confirmation of the impaired NF‐κB signaling pathway upon stable A3C knockdown. Fig. S4. Clinical relevance of NF‐κB family members and NF‐κB target genes in RCC. Fig. S5. Binding partners of A3C belong to diverse HALLMARK gene sets. Fig. S6. A3C depletion in 769‐P results in reduced expression of NF‐κB signaling pathway regulators and impaired nuclear translocation of NF‐κB subunits. Fig. S7. A3C regulates cell viability under diverse growth conditions and drug treatment. [file MOL2-19-114-s003.zip › Supplemental_Figures.pdf]

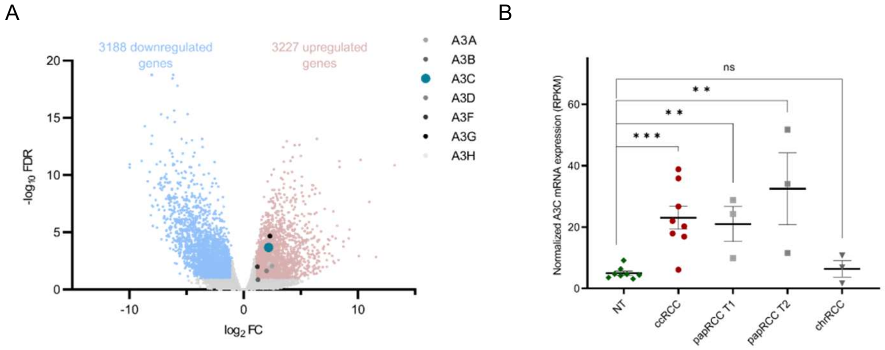

Supplemental Figure 1. Expression of A3 family members in a separate, small RCC cohort.

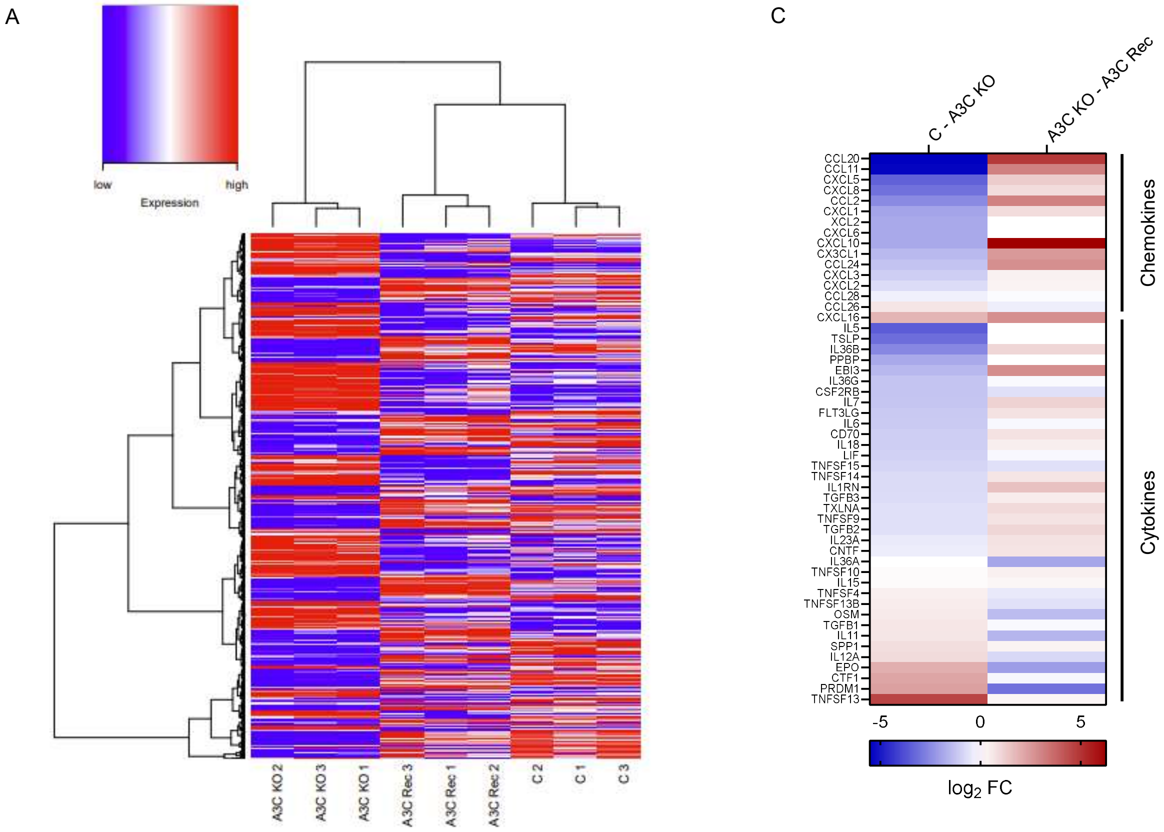

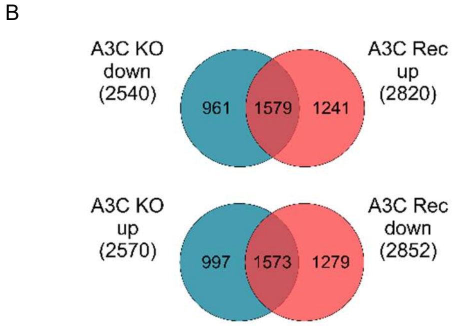

Supplemental Figure 2. RNA-seq in 786-O CRISPR/Cas9-mediated A3C KO and A3C Rec cells.

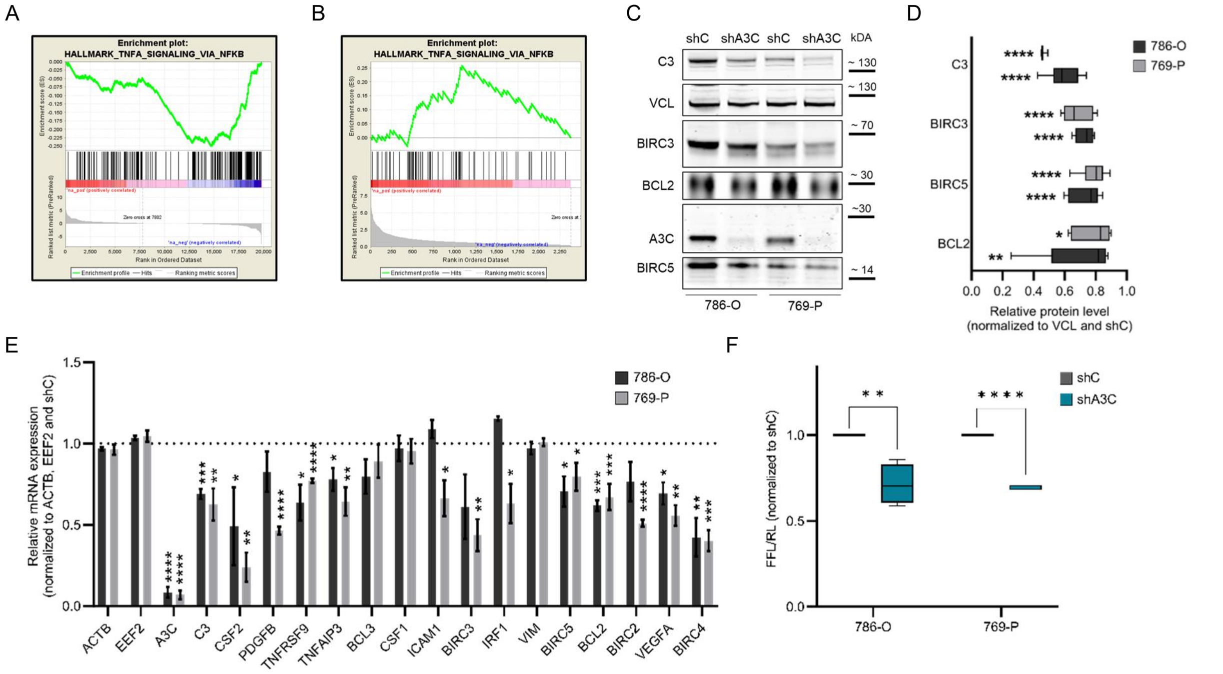

Supplemental Figure 3. Confirmation of the impaired NF-κB signaling pathway upon stable A3C knockdown.

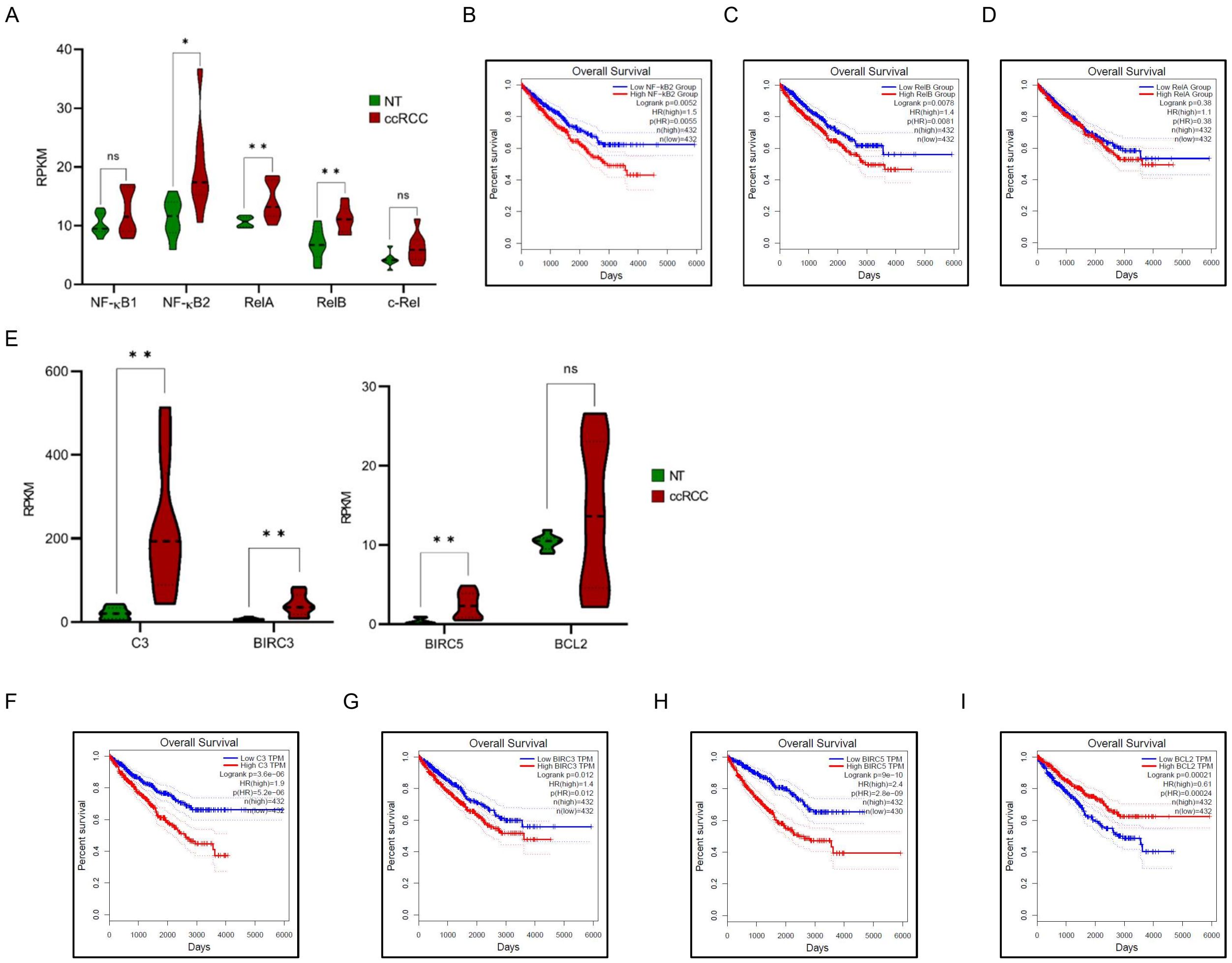

Supplemental Figure 4. Clinical relevance of NF-κB family members and NF-κB target genes in RCC.

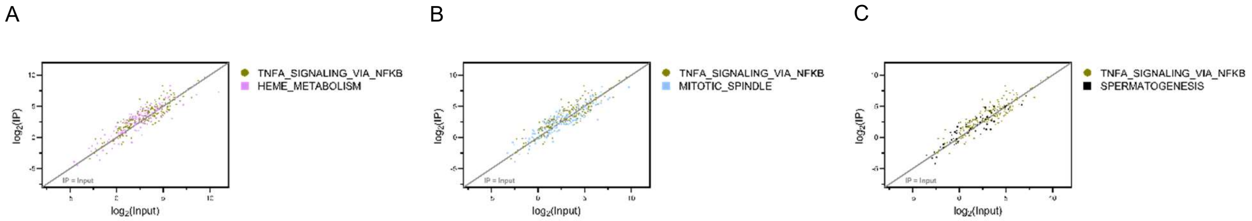

Supplemental Figure 5. Binding partners of A3C belong to diverse HALLMARK gene sets.

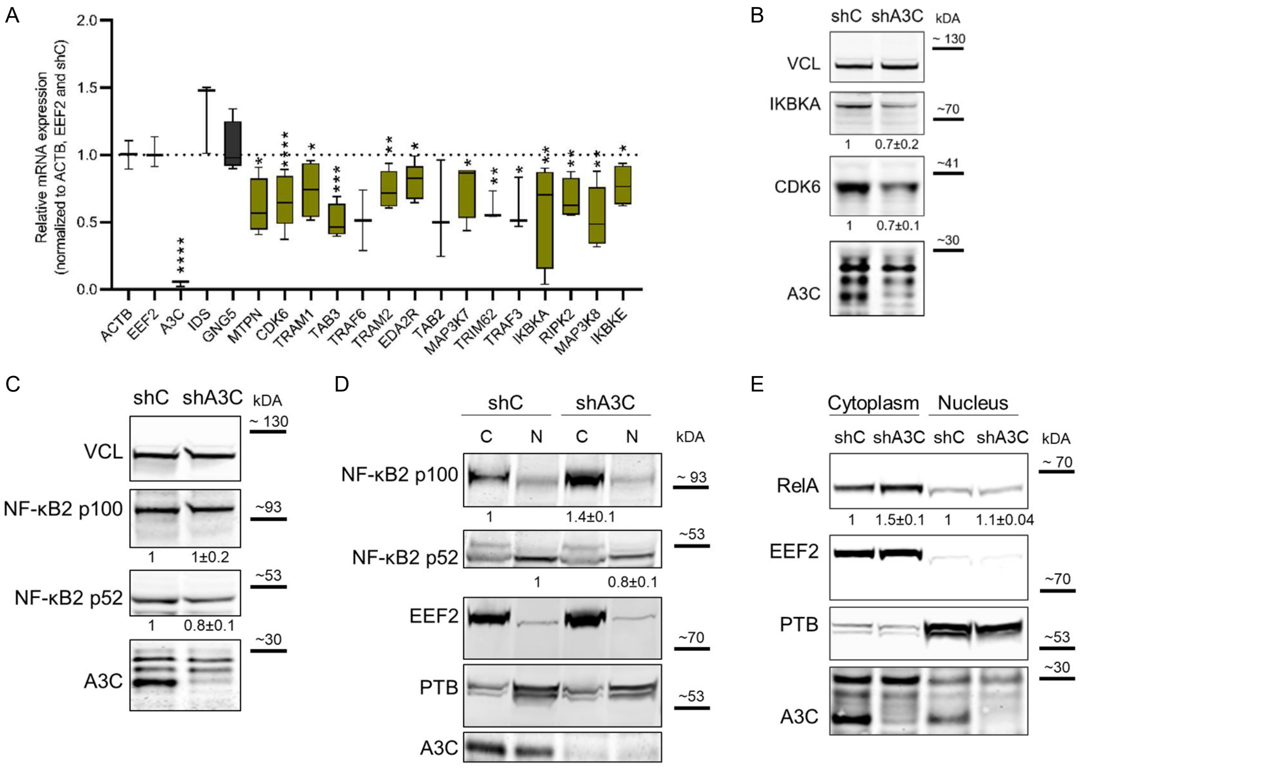

Supplemental Figure 6. A3C depletion in 769-P results in reduced expression of NF-κB signaling pathway regulators and impaired nuclear translocation of NF-κB subunits.

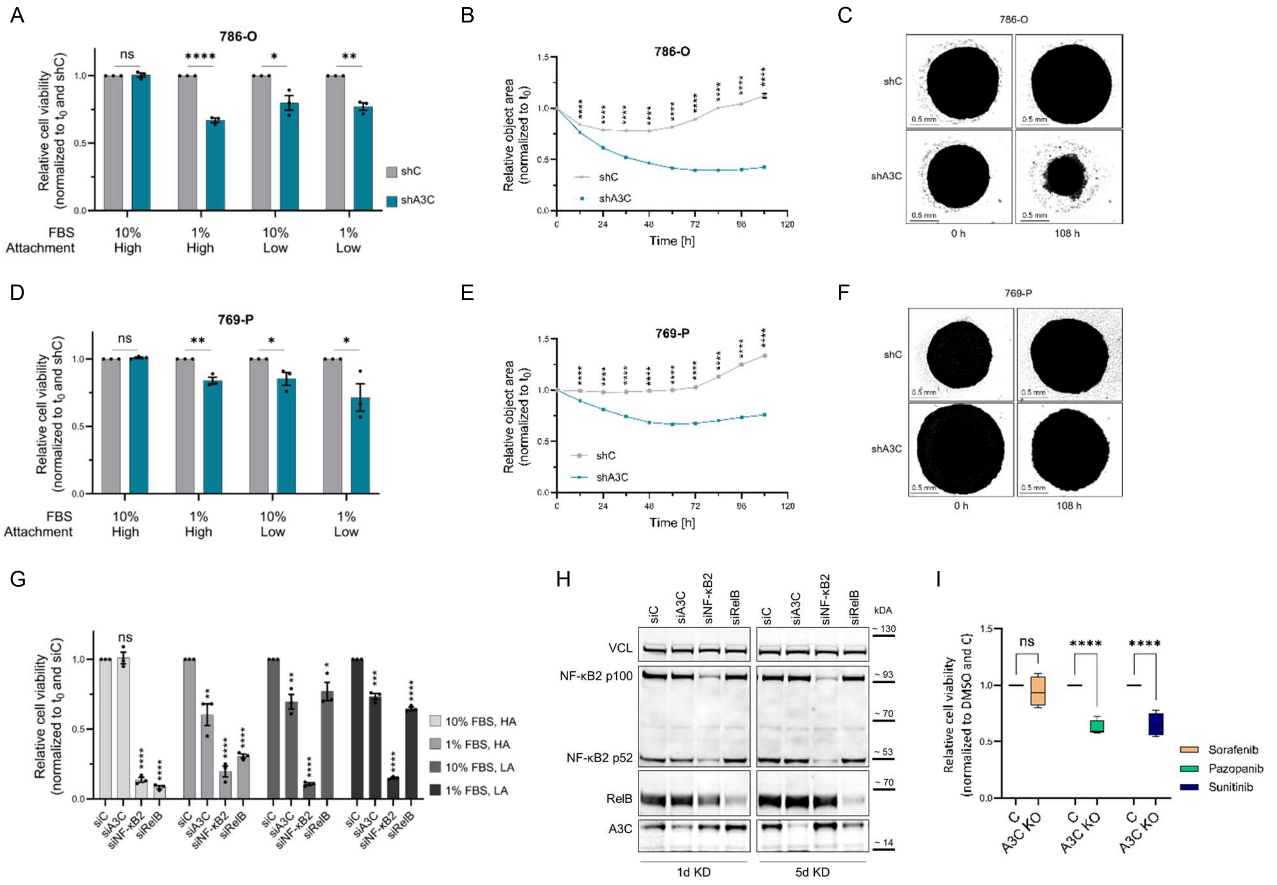

Supplemental Figure 7. A3C regulates cell viability under diverse growth conditions and drug treatment.
